# Leveraging ECG foundation models in critical care for sinus rhythm and atrial fibrillation classification

**DOI:** 10.1186/s40635-026-00949-7

**Published:** 2026-08-26

**Authors:** Maria Galanty, Michiel Hulleman, Merijn C. Reuland, Björn van der Ster, Alexander P. J. Vlaar, Clara I. Sánchez, the AF Expert Panel

**Affiliations:** 1 Informatics Institute, University of Amsterdam (UvA), Amsterdam, Netherlands; 2 Department of Biomedical Engineering and Physics, Amsterdam University Medical Center (Amsterdam UMC), Amsterdam, Netherlands; 3 Department of Anaesthesiology, Amsterdam UMC, Amsterdam, Netherlands; 4 Department of Intensive Care, Amsterdam UMC, Amsterdam, Netherlands; 5 Department of Clinical and Experimental Cardiology, Amsterdam UMC, Amsterdam, Netherlands; 6 Amsterdam Cardiovascular Sciences, Amsterdam UMC, Amsterdam, Netherlands

**Keywords:** ICU, ECG, Deep learning, Atrial fibrillation, Continuous monitoring

## Abstract

****Background**:**

Recent advances in deep learning have led to the development of ECG foundation models (ECG-FMs) trained with self-supervised learning, which can extract generalizable representations from large-scale data. In this study, we evaluated the performance of these ECG-FMs in detecting atrial fibrillation and sinus rhythm during continuous monitoring of intensive care unit (ICU) patients, using data from Amsterdam University Medical Center (Amsterdam UMC). Our three-stage study: (i) used within-dataset classification performance on public PhysioNet datasets as an indirect proxy for label consistency, (ii) tested off-the-shelf fine-tuned ECG-FMs on ICU data, and (iii) fine-tuned ECG-FM on curated public datasets, with and without weakly labelled in-house ICU data.

****Results**:**

Off-the-shelf model showed limited transferability (F1 = 0.68 for sinus rhythm (SR), 0.52 for atrial fibrillation (AF)). Fine-tuning on consistent public datasets achieved robust performance (F1 = 0.91 for SR, 0.82 for AF), while adding weakly labelled ICU data further improved recall at some cost to precision. Performance remained stable across varying ECG lead configurations, supporting adaptability to heterogeneous inputs.

**Conclusions:**

These findings indicate that off-the-shelf pre-trained ECG-FMs do not generalise well to continuous monitoring in ICU populations; however, after targeted fine-tuning, they can achieve strong performance even in unseen cohorts, underscoring the importance of high-quality fine-tuning data and rigorous local validation.

## Introduction

Atrial fibrillation (AF) is a common arrhythmia characterised by disorganised atrial activity and irregular heartbeats [1]. In the intensive care unit (ICU), where patients often have complex conditions, AF is especially prevalent and linked to worse outcomes, including stroke, heart failure, and mortality [2–4]. Electrocardiography (ECG) is used for arrhythmia diagnosis. Detecting AF during continuous monitoring of critically ill patients is challenging due to rapidly changing physiology, overlapping conditions [2], as well as patients’ movements and artefacts introduced during the delivery of medical care. While the ICU environment enables rapid recognition of life-threatening events, it also introduces additional analytical complexity, necessitating validation against ECG data collected during routine clinical evaluations or ambulatory monitoring.

Automated AF detection has the potential to streamline diagnosis, enable earlier intervention, and ultimately improve patient outcomes. However, it remains challenging in the ICU due to domain shifts between training and clinical data, as well as challenges in the creation of labelled data for training. Numerous deep learning approaches have been proposed for arrhythmia detection, yet their performance often degrades when applied beyond their training domain [5]. This limitation may arise from insufficient model generalizability or data-related issues, as deep learning frameworks depend on large labelled datasets. While in-house labelling ensures data quality, it is time-consuming and requires expertise, whereas publicly available ECG datasets often suffer from inconsistent, heterogeneous, or semi-automated annotations, leading to noisy and unreliable labels [5]. Moreover, most of the arrhythmia detection studies have focused on non-ICU populations using ambulatory monitor data recorded during patients’ daily activities [6–8]. The ICU population, however, presents distinct physiological characteristics which may additionally limit the transferability of such models. Some approaches (e.g., [9, 10]) utilized the MIMIC-IV-ECG subset [11], which comes from ICU population but includes diagnostic ECG and machine-generated rather than expert-verified annotations. Recent work incorporating well-labelled ICU data, such as by Chen et al. [12, 13], has shown moderate AF detection performance.

New frontiers in deep learning have introduced ECG foundation models (ECG-FMs) that leverage self-supervised learning to extract generalizable representations directly from large-scale data [14–17]. These models are promising as they learn intrinsic data features without extensive manual annotation and can subsequently be fine-tuned for specific downstream tasks. However, whether such approaches can effectively address the unique challenges of ICU rhythm classification remains an open question.

To explore this, we conducted a multi-stage experimental study evaluating ECG-FMs for AF and sinus rhythm (SR) classification in the ICU. Specifically, we (i) used within-dataset classification performance as an indirect proxy for label consistency across publicly available PhysioNet Challenge 2021 data [5], (ii) evaluated out-of-the-box foundation model on labelled ICU data from Amsterdam University Medical Center (Amsterdam UMC), and (iii) investigated whether fine-tuning on curated diagnostic ECG public datasets, with or without additional institution-specific weakly labelled data, could improve generalizability and robustness. We hypothesise that ECG-FMs, pre-trained on diverse datasets, offer greater robustness to domain shifts and variable ECG lead configurations. Ultimately, this work aims to assess their potential as scalable, clinically meaningful tools for arrhythmia detection in critically ill patients.

## Methods

### Data

In this study, we utilised two primary sources of data - the PhysioNet Challenge 2021 repository [5, 18] and the internal dataset collected at the Amsterdam UMC. Our specific focus in this project is to detect whether an ECG sample corresponds to AF or to SR, where the latter includes sinus tachycardia, sinus bradycardia, and normal SR.

#### Physionet challenge dataset

For this project, we sourced data from the PhysioNet Challenge 2021 [5, 18]. This repository integrates data from multiple institution across continents, acquired in hospitals and clinical settings, including the China Physiological Signal Challenge in 2018 (CPSC 2018) and its additional version (CPSC-Extra) [19], the St Petersburg INCART 12-lead Arrhythmia Database (INCART) [20], the Physikalisch-Technische Bundesanstalt databases (PTB and PTB-XL) [21, 22], the Georgia 12-lead ECG Challenge Database, as well as collections from Chapman University, Shaoxing People’s Hospital (Chapman-Shaoxing), and Ningbo First Hospital (Ningbo) datasets [23, 24]. The datasets were collected from a range of institutions, each employing distinct annotation methods. According to the Challenge organisers, this leads to real-world heterogeneity, with varying prevalence of cardiac abnormalities across databases and the presence of labelling errors. In several cohorts, annotations were generated by machine algorithms and subsequently reviewed by one or more human experts, resulting in variable label quality. In total, the datasets contained 133 diagnostic categories, which were consolidated through merging, relabeling, or exclusion into 30 categories for use in the Challenge.

Our project is specifically focused on detecting AF and SR. To ensure sufficient representation of these classes, we excluded datasets with too few (below 200) AF or SR examples (Ningbo, INCART, CPSC-Extra, and PTB), as training on severely imbalanced datasets is inherently more challenging and an adequate number of examples per class is necessary to meaningfully assess label consistency. We then concentrated our analysis on four publicly available cohorts with adequate class representation namely, PTB-XL, Chapman-Shaoxing, Georgia, and CPSC.

#### Amsterdam UMC dataset

At the Amsterdam UMC, ECG signals are continuously recorded and stored in the institutional data warehouse, as described by Noteboom et al. [25]. However, the stored data contains only a subset of the leads measured from patients, with some leads missing, as the warehouse captures only the leads selected for display on the bedside monitor by clinical staff. In parallel, ICU nursing staff document heart rhythm observations on an hourly basis in the electronic health record (EHR) system, categorising them into 16 predefined types. For this study, we leveraged both data sources, the continuous ECG recordings and the nurse-documented rhythm annotations to curate two ECG datasets from the Amsterdam UMC (Amsterdam UMC Expert and Amsterdam UMC-weak datasets), derived from anonymized data extracted from the institutional data warehouse. (Medical Ethics Review Committee Amsterdam UMC: study not subject to WMO; approval not required, Ref. No. W23_031#23.054).

***Amsterdam UMC-weak dataset construction***

First, for model development, we constructed a weakly labelled dataset by aligning each nursing recorded rhythm observation with the 10-second ECG segment within a maximum matching window of one minute between the annotation timestamp and the ECG recording time. We note that rhythm observations in our Epic EHR are typically charted every hour, introducing temporal uncertainty between the documented and true time of rhythm assessment. Segments showing missing signals or constant flatlines in all leads were excluded, as they contained no useful data. In this case, the labels are considered weak because they originate from routine clinical documentation rather than expert adjudication of the ECG signals. We extracted 8,500 ten-second ECG segments from 119 patients, using the nurses’ rhythm observation notes as labels to form the weakly labelled dataset for model training. This dataset is referred to as the Amsterdam UMC-weak dataset.

***Amsterdam UMC expert dataset construction***

Next, we constructed a second weakly labelled dataset, applying the same alignment procedure. To prevent patient overlap and mitigate the risk of data leakage between training and evaluation, we restricted this dataset to a different cohort of patients by selecting a separate time frame for data extraction. The dataset consisted of 25,714 samples from 349 patients. To create a high-quality reference dataset, we sampled 600 ECG segments from this larger set. Sampling was stratified to preserve the original distribution of the 16 rhythm categories documented in clinical practice by nurses, ensuring representation across a broad range of conditions. To maintain this distribution while including as many patients as possible from the original cohort, we applied a randomised sampling strategy that balanced category proportions with patient diversity. These 600 segments were subsequently reviewed and re-annotated by an expert panel to establish a reliable reference standard. This dataset is referred to as Amsterdam UMC Expert.

The annotation process was completed by the AF Expert Panel. Each 10-second segment was bandpass-filtered (1–47 Hz) using a zero-phase third-order Butterworth filter to remove noise while preserving physiologically relevant signals [26]. Next, six physicians trained in ECG interpretation served as annotators. The 600 ECG segments were divided into six batches of 100 segments each. Every batch was assigned to a distinct pair of physicians, such that each segment was independently reviewed by two annotators. This design ensured that every annotator was paired with two colleagues, allowing assessment of inter-rater variability across different rater combinations. The annotation protocol followed a structured, two-step procedure. First, reviewers assessed the signal quality, categorising each segment as clean, noisy but interpretable, or too noisy to label. Segments marked as too noisy were excluded from further analysis. Second, they assigned one or more rhythm categories, choosing from SR (including normal SR, sinus bradycardia, and sinus tachycardia), AF, or Other (e.g., atrial flutter, ventricular tachycardia, or pacemaker rhythm). Reviewers were also able to provide free-text comments to capture any additional observations about the segment. Segments were referred to a senior cardiology clinician in any of the following situations: (i) the two annotators disagreed on the assignment of noise level 2 (too noisy to label); (ii) the annotators disagreed on whether SR or AF was present in the segment; or (iii) both junior annotators expressed uncertainty about their judgment. In these cases, the senior clinician provided the final adjudicated label. Inter-rater agreement was therefore assessed exclusively for SR and AF labels. Annotations for the Other rhythm category were used only as supporting contextual information (e.g., when evaluating potential misclassifications).

#### Data preprocessing

All data used in this study were preprocessed following the approach described by Nejedly et al. [27]. First, all ECG signals were upsampled to a uniform sampling rate of 500 Hz when necessary. Second, a zero-phase third-order Butterworth bandpass filter [26] was applied with cut-off frequencies of 1 Hz and 47 Hz. Finally, z-score normalisation was performed to standardise each ECG segment across all samples. Upsampling and z-score normalisation were performed to ensure compatibility with the ECG-FM model and to unify data originating from diverse sources.

### ECG-FM

In this study, we employed as ECG-FM an open-source transformer-based architecture [28] pretrained on approximately 1.5 million ECGs from the PhysioNet Challenge 2021 dataset [5] and the MIMIC-IV-ECG dataset [11]. The development of the ECG-FM builds on a previous work [29], which introduced Random Lead Masking (RLM) as a strategy to improve robustness in handling downstream tasks with arbitrary subsets of ECG leads. The architecture is optimised for fixed-length ECG segments of 5 seconds, providing a standardised input format for downstream applications. Because the diagnostic information in an ECG depends on the number and spatial arrangement of leads, model performance is inherently tied to the lead configuration used at inference. ECG-FM is pretrained with RLM, where individual leads are randomly dropped, so the backbone is explicitly exposed to many partial-lead configurations and can be evaluated with reduced-lead inputs without modifying the architecture, while also improving robustness to varying input lead configurations and issues related to e.g., occasional electrode disconnections.

We accessed a publicly available fine-tuned version of ECG-FM via Hugging Face [30], trained on the MIMIC-IV-ECG dataset for 17 rhythm classes (accessed July 2025). Although the original model was fine-tuned using both MIMIC-IV-ECG and a private dataset, only the weights derived from MIMIC-IV data are publicly released due to privacy constraints.

#### Experiment 1: public dataset selection and label consistency assessment

This experiment examines the annotation consistency of publicly available PhysioNet Challenge datasets, which organisers have previously noted that label quality may vary across datasets [5], a concern reinforced by later studies on public ECG databases [31]. They also reported substantial performance differences across test databases, highlighting dataset generalizability as a key challenge [18].

To investigate these issues without relying on manual expert review, we adopted an indirect strategy by training classifiers on each dataset independently. In this experiment, we specifically evaluated the consistency of rhythm classification for AF and SR within individual datasets. We hypothesised that higher test performance on data drawn from the same source as the training and validation sets would indicate more reliable labelling and reduced variability in intra- and inter-annotator agreement [32].

For each dataset (PTB-XL [22], Chapman-Shaoxing [24], Georgia, and CPSC [33]), ECG-FM was fine-tuned by appending two linear classification layers with non-exclusive outputs for AF and SR. Because ECG-FM operates on 5-second windows, all ECGs were segmented accordingly, with random windows sampled for public datasets. Fine-tuning was performed separately on each dataset using stratified 5-fold cross-validation. In each iteration, three folds were used for training, one for validation (for early stopping and threshold optimisation), and one as a held-out test subset. Performance was averaged across folds to provide a robust estimate of within-dataset consistency. Consistently high performance served as a proxy for reliable labelling.

Model optimisation was performed using the Adam optimiser [34]. The initial learning rate was set to 0.001 and reduced by a factor of 0.1 (up to two times) when validation loss failed to improve for five consecutive epochs. Training was terminated early if no improvement was observed for five epochs after the final learning rate adjustment, with a maximum training duration of 100 epochs. A batch size of 128 was used for all experiments. Models were implemented in Python using the PyTorch framework.

### Experiment 2: ECG-FM evaluation

In this second experiment, we evaluated the out-of-the-box performance of ECG-FM on the Amsterdam UMC Expert dataset. The goal of this analysis was to establish a baseline understanding of how well publicly available fine-tuned version of the model generalise to unseen, institution-specific data without any additional adaptation. For this purpose, we evaluated the fine-tuned version of ECG-FM hosted on Hugging Face [30].

### Experiment 3: ECG-FM fine-tuning

In this experiment, we first investigated the effect of fine-tuning ECG-FM on selected public datasets that showed more consistent labelling for AF and SR classes in Experiment 1. We used these curated datasets to fine-tune ECG-FM for multi-label classification of AF and SR. We further examined whether including our weakly annotated Amsterdam UMC ICU dataset (Amsterdam UMC-weak), despite its lower reliability, could improve performance by introducing domain-specific variability not captured in public datasets. Fine-tuning was conducted under two regimes: (i) Public (Pub) – using only the selected high-quality public datasets, and then (ii) the public datasets combined with the Amsterdam UMC in-house weakly labelled ICU dataset (Pub + Amsterdam UMC-weak).

The fine-tuning setup was identical to that described in Section 2.2.1. For model adaptation, the ECG-FM architecture was extended with two linear classification layers that produced non-exclusive outputs for AF and SR. Since ECG-FM processes 5-second input windows, each ECG was segmented accordingly. For the public datasets, random 5-second segments were sampled, whereas for the Amsterdam UMC dataset, the first 5 seconds of each 10-second annotated segment were used. Training employed a binary cross-entropy loss function. Public datasets and Amsterdam UMC-weak were split into training (70%), validation (20%), and test (10%) sets, with classification thresholds optimised on the validation set by maximising F1 score.

### Evaluation methods

Model performance was assessed using standard classification metrics, including accuracy, precision, recall, and F1-score. The Amsterdam UMC Expert dataset was designed to reflect the diversity and proportions of ICU patients in terms of cardiac rhythm and demographics. However, the dataset is not balanced with respect to AF and SR labels. This imbalance highlights the importance of employing multiple evaluation metrics rather than relying solely on accuracy.

During fine-tuning (Experiment 1 and 3), each regime was repeated with five random seeds to ensure reliability, and results were averaged across runs. Differences between models trained with or without weakly labelled data as well as subgroup differences were evaluated bootstrap 95% confidence intervals (CI), computed by resampling test patients with replacement (10,000 resamples per run, pooled across seeds). Cliff’s delta was reported as a non-parametric effect size to quantify the consistency of differences across seeds, with non-overlapping CIs indicating a practically meaningful difference. For the third experiment, performance was further stratified by sex and age. To additionally test robustness within this experiment, we repeated the evaluation on the Amsterdam UMC Expert set using the second half of the 10-second ECG recordings (i.e., the 5–10s segments).

## Results

### Experiment 1: public dataset selection and label consistency assessment

**Fig. 1:**
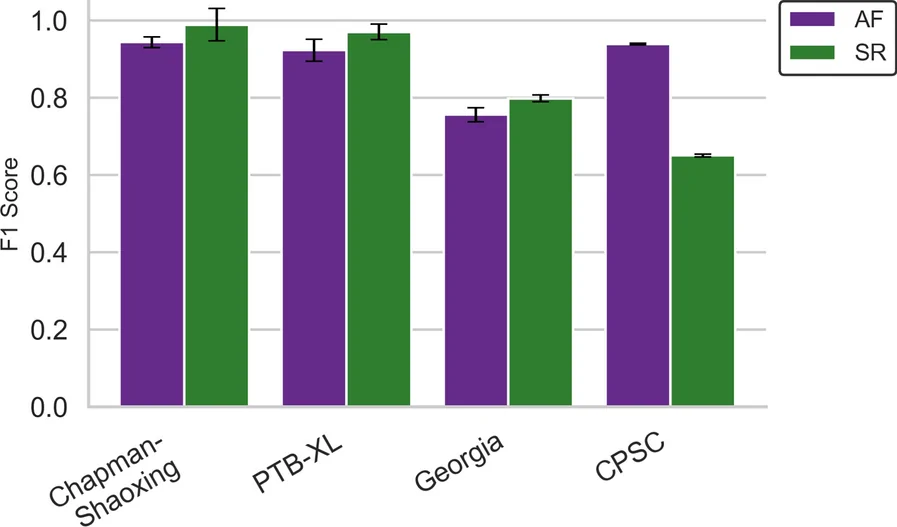
Experiment 1 results: Mean F1 scores with 95% confidence intervals across four public datasets (Chapman-Shaoxing, PTB-XL, Georgia and CPSC)

**Table 1:** Experiment 1 results: Internal label consistency of four public ECG datasets

**Dataset**	**Class**	**Accuracy**	**Precision**	**Recall**	**F1**
PTB-XL	AF	0.989±0.001	0.918±0.014	0.929±0.020	0.923±0.007
	SR	0.948±0.004	0.950±0.004	0.992±0.005	0.971±0.003
Chapman-Shaoxing	AF	0.980±0.005	0.914±0.025	0.975±0.005	0.944±0.013
	SR	0.984±0.002	0.985±0.002	0.993±0.003	0.989±0.001
Georgia	AF	0.975±0.004	0.803±0.056	0.716±0.018	0.756±0.030
	SR	0.809±0.015	0.763±0.016	0.837±0.020	0.799±0.015
CPSC	AF	0.979±0.004	0.948±0.014	0.931±0.019	0.939±0.010
	SR	0.903±0.009	0.628±0.021	0.675±0.030	0.651±0.021

For each dataset, ECG-FM was fine-tuned using 5-fold cross-validation to classify SR and AF. Metrics (accuracy, precision, recall, F1) are averaged across folds

Table 1 summarises the performance of ECG-FM models fine-tuned and evaluated separately on each of the four selected public datasets (PTB-XL, Chapman-Shaoxing, Georgia, and CPSC). For each dataset, classification results for AF and SR are reported as the mean accuracy, precision, recall, and F1 score across the five cross-validation folds. We also visualise the corresponding F1 scores for AF and SR detection from Experiment 1 in Figure 1. When examining F1 scores, PTB-XL and Chapman–Shaoxing consistently outperformed the Georgia and CPSC datasets for both AF and SR. Therefore, PTB-XL and Chapman–Shaoxing were selected for use in Experiment 3 to fine-tune ECG-FM.

**Fig. 2:**
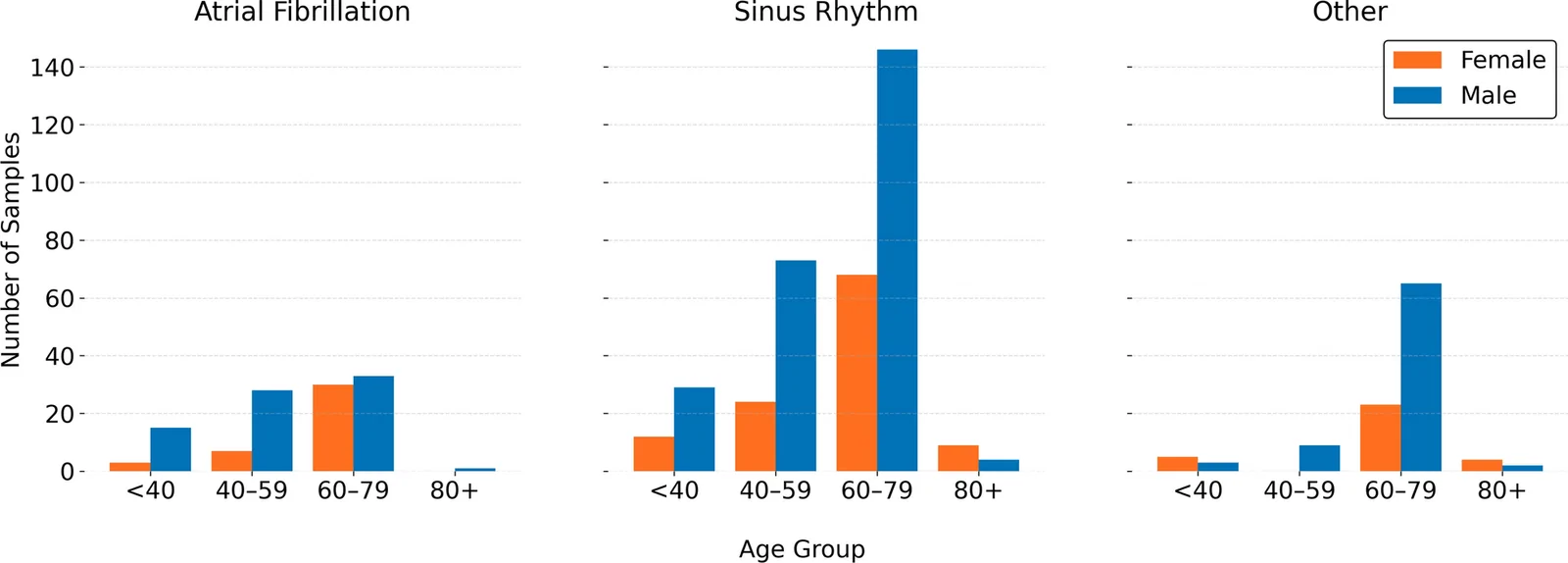
Distribution of samples in the Amsterdam UMC Expert dataset by diagnostic category. Each panel corresponds to one category: AF (left), SR (middle), and Other (right). Bars show the number of female (orange) and male (blue) samples across four predefined age groups (<40, 40–59, 60–79, 80+ years)

**Fig. 3:**
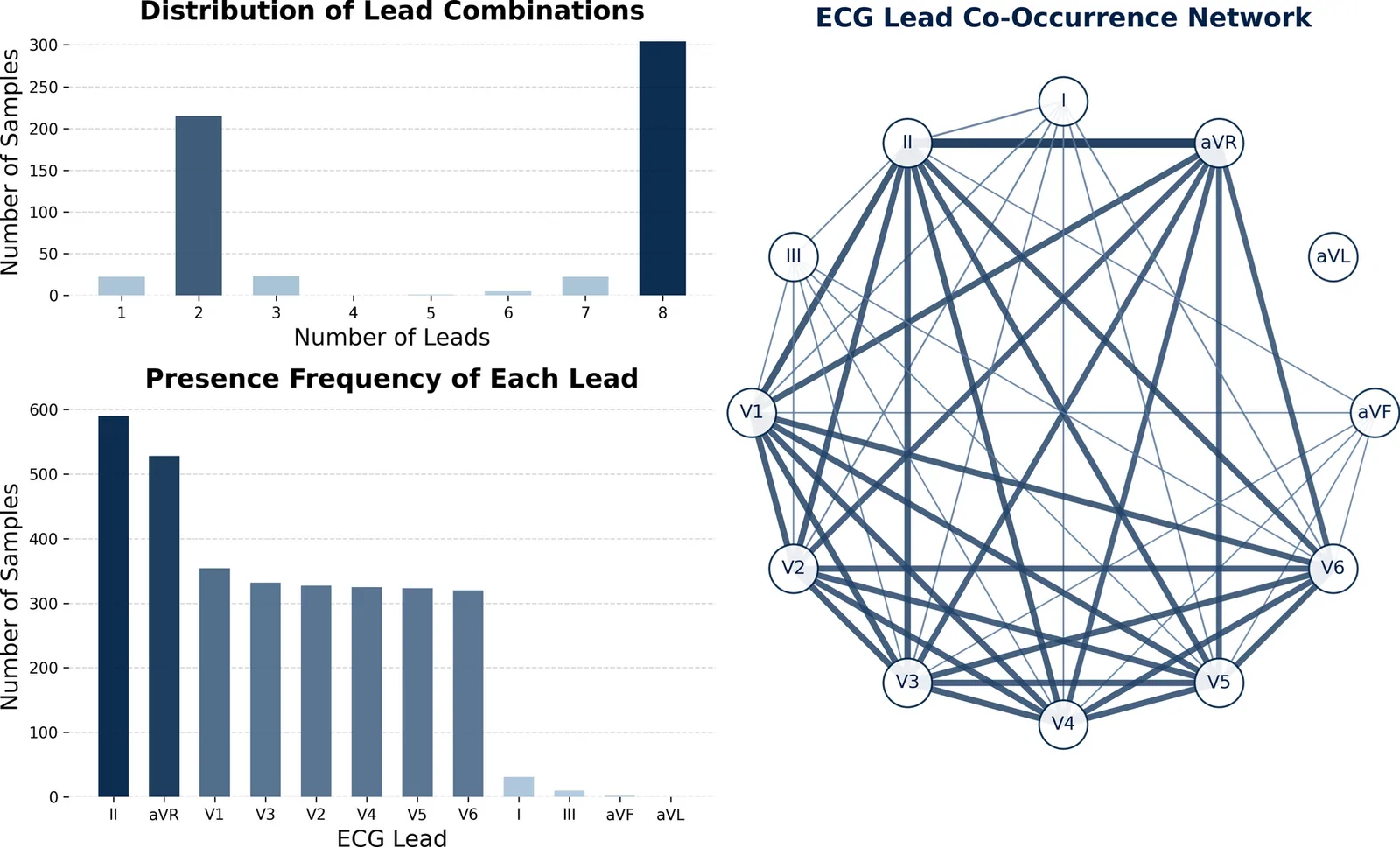
Amsterdam UMC Expert overview. Left (top): distribution of the number of available leads per sample. Left (bottom): frequency of occurrence of each individual ECG lead across the dataset. Right: co-occurrence network of ECG leads, where edges represent the number of times two leads are simultaneously available in the same sample (thicker edges correspond to more frequent co-occurrence)

**Table 2:** Experiment 2 results: Classification performance of publicly available fine-tuned ECG-FM on the Amsterdam UMC test set for SR and AF

**Fine-Tuning Datasets**	**Class**	**Accuracy**	**Precision**	**Recall**	**F1**
MIMIC-IV-ECG	AF	0.664	0.359	0.965	0.523
	SR	0.557	0.610	0.762	0.677

The left column shows the fine-tuning dataset

**Table 3:** Experiment 3 results: Classification performance of two ECG-FM models fine-tuned using only public data (Pub) and using both public and Amsterdam UMC-weak data (Pub+Amsterdam UMC-weak)

**Fine-Tuning Regime**	**Class**	**Accuracy**	**Precision**	**Recall**	**F1 Score**
**Public**					
Pub	AF	0.987±0.000	0.921±0.009	0.953±0.011	0.937±0.002
	SR	0.962±0.001	0.959±0.002	0.996±0.001	0.977±0.001
Pub + Amsterdam UMC-weak	AF	0.985±0.001	0.898±0.008	0.968±0.006	0.932±0.002
	SR	0.961±0.001	0.959±0.002	0.995±0.001	0.977±0.001
**Amsterdam UMC Weak**					
Pub	AF	0.945±0.004	0.715±0.042	0.779±0.058	0.743±0.017
	SR	0.928±0.003	0.971±0.004	0.944±0.006	0.957±0.002
Pub + Amsterdam UMC-weak	AF	0.964±0.005	0.805±0.054	0.858±0.040	0.829±0.012
	SR	0.948±0.004	0.965±0.006	0.975±0.008	0.970±0.002
**Amsterdam UMC Expert**					
Pub	AF	0.930±0.006	0.867±0.026	0.770±0.052	0.814±0.021
	SR	0.894±0.005	0.917±0.010	0.911±0.012	0.914±0.004
Pub + Amsterdam UMC-weak	AF	0.934±0.006	0.855±0.045	0.809±0.025	0.830±0.009
	SR	0.899±0.003	0.903±0.008	0.938±0.010	0.920±0.003

Results are averaged across five random seeds; standard deviation is given after ±

### Local dataset characteristics

The Amsterdam UMC Expert dataset initially comprised 600 ECG segments, which were annotated by experts. Eight segments were excluded due to poor quality, leaving 592 validated samples from 226 unique patients. Based on expert consensus, 365 segments (62%) were labelled as SR, 117 (20%) as AF (including one sample labelled as both AF and SR (see Appendix Figure 6), and 111 (18%) as other rhythms (i.e., segments that did not receive either an AF or an SR label). Table 5 and 6 in Appendix provide further details on each class sample count for Amsterdam UMC and public datasets. In addition, we evaluated the Positive Predictive Value (PPV) of nurse-assigned labels against clinician-annotated rhythm labels, obtaining a PPV of 0.91 for AF and 0.77 for SR (see Appendix Fig. 7). A detailed summary of nurse-assigned rhythm labels for samples whose ground truth is neither AF nor SR is provided in Appendix Table 7. Inter-rater agreement among the six annotators was assessed using Cohen’s Kappa coefficient separately for SR and AF. Pairwise kappa among annotators had a median of 0.60 for SR and 0.49 for AF. As expected, agreement between annotators and the senior annotator yielded a lower median kappa (0.35) for both SR and AF, as this subset consisted exclusively of cases where annotators disagreed (see Appendix  Fig. 8, Fig. 9 and Table 8). Similar to Experiment 1, we assessed label consistency for the Amsterdam UMC datasets; see Appendix Tables 9 and 10.

Figure 2 summarises the demographic composition of the samples in the Amsterdam UMC Expert cohort. Samples were stratified by the rhythm diagnosis and grouped across four age categories (<40, 40–59, 60–79, 80+ years) and sex. The number and type of available ECG leads varied substantially across samples. Figure 3 illustrates the lead distribution and co-occurrence patterns within the Amsterdam UMC Expert dataset. Most recordings contained either two or eight leads. Leads II and aVR were the most frequently available, whereas precordial leads (V1–V6) appeared less consistently.

### Experiment 2: ECG-FM evaluation

The second experiment examined the out-of-the-box performance of the publicly available fine-tuned ECG-FM on Amsterdam UMC Expert. Results are presented in Table 2. Metrics are provided separately for AF and SR classification.

**Fig. 4:**
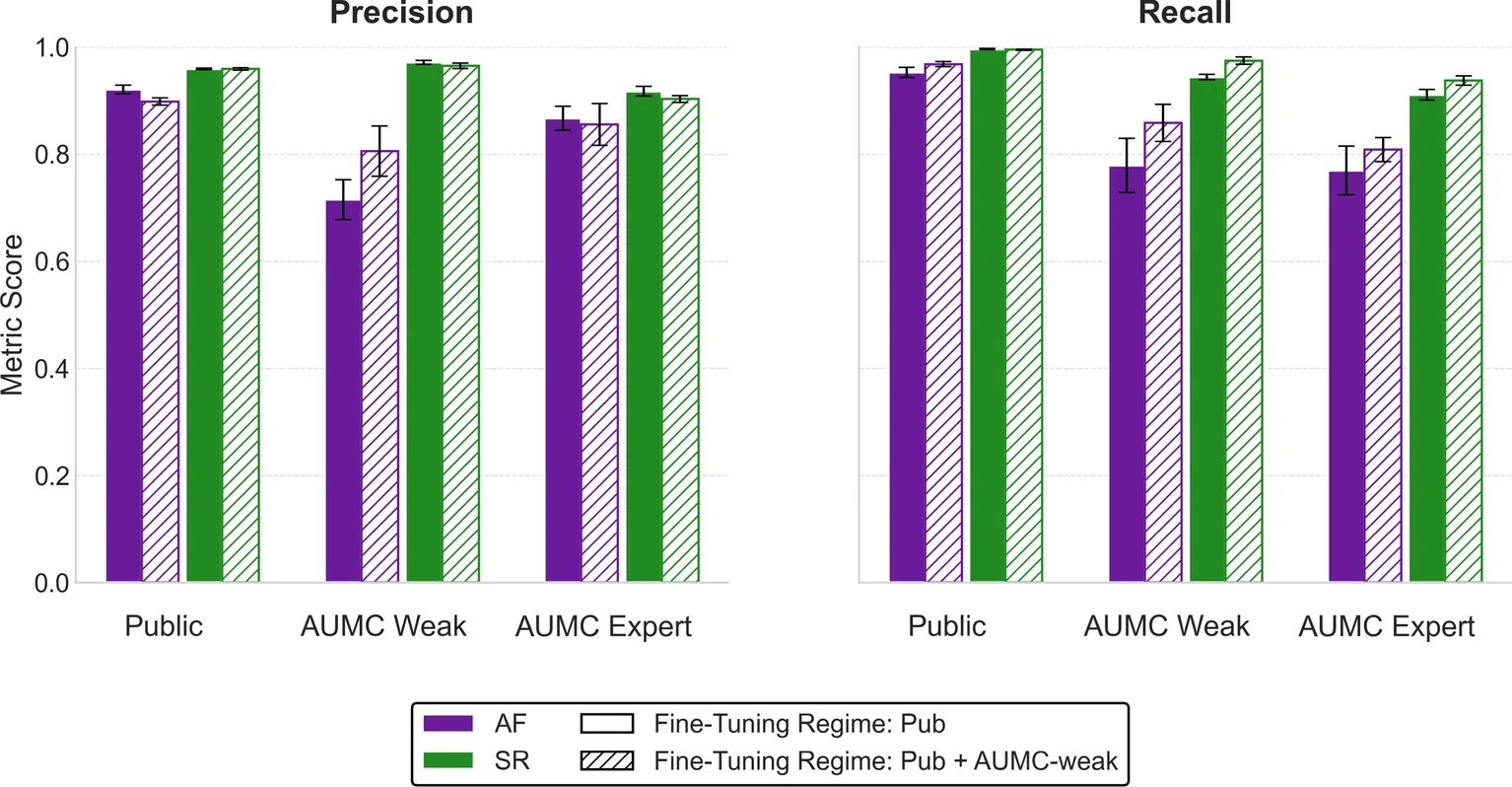
Experiment 3 results: Precision and recall for AF and SR across three evaluation datasets (Public test, Amsterdam UMC-weak, Amsterdam UMC Expert) for two ECG-FMs fine-tuned in this study. Bars represent models fine-tuned without Amsterdam UMC-weak data (Pub), and dashed lines represent models fine-tuned with Amsterdam UMC-weak data (Pub+Amsterdam UMC-weak). Error bars indicate 95% confidence intervals

**Table 4:** Experiment 3 results: Classification performance of two fine-tuned ECG-FMs, one on public data only (Pub) and one on public plus Amsterdam UMC-weak data (Pub+Amsterdam UMC-weak), reported for SR and AF on the second half of the Amsterdam UMC Expert test set (5–10 s segments), averaged across random seeds

**Fine-Tuning Regime**	**Class**	**Accuracy**	**Precision**	**Recall**	**F1**
Pub	AF	0.925 (–0.005)	0.855 (–0.013)	0.752 (–0.017)	0.799 (–0.015)
	SR	0.887 (–0.007)	0.911 (–0.006)	0.905 (–0.006)	0.908 (–0.006)
Pub + Amsterdam UMC-weak	AF	0.934 (–0.000)	0.851 (–0.004)	0.812 (+0.004)	0.830 (–0.000)
	SR	0.894 (–0.005)	0.894 (–0.009)	0.940 (+0.003)	0.916 (–0.004)

Values in parentheses show the difference from the first-half test set

**Fig. 5:**
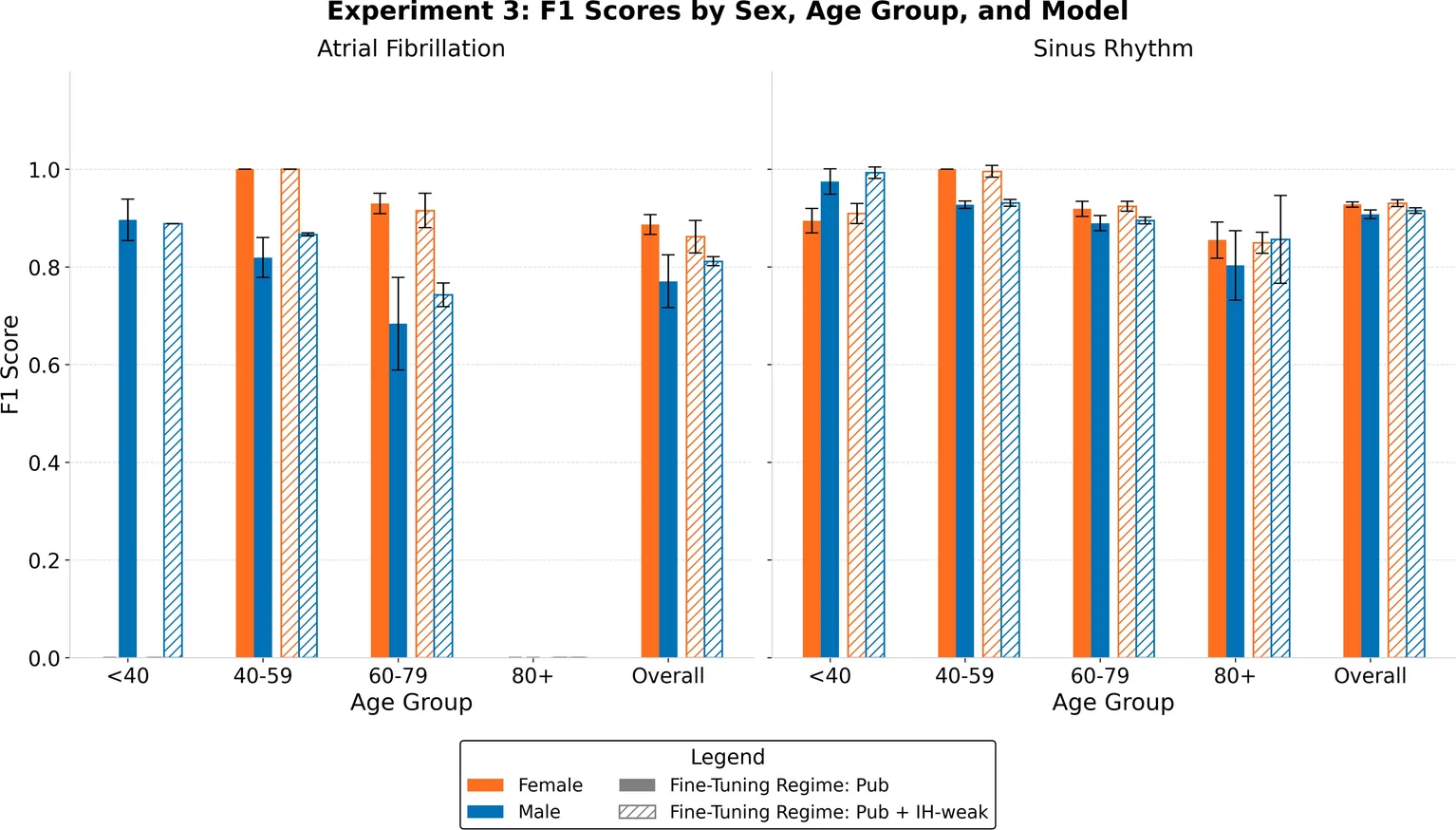
Experiment 3 results: F1 scores for AF and SR stratified by target. Results are shown for Amsterdam UMC Expert models fine-tuned without (solid) and with (hatched) weakly labelled Amsterdam UMC data. Bars indicate means over five runs with 95% CIs. Groups are by sex (orange = female, blue = male), age (<40, 40–59, 60–79, 80+), and overall. Asterisks mark significant sex differences within age groups (*P ≤ 0.05*, Mann–Whitney U test)

### Experiment 3: ECG-FM fine-tuning

The third experiment investigated the effect of fine-tuning ECG-FM with selected public datasets (PTB-XL and Chapman-Shaoxing) alone or in combination with the weakly labelled Amsterdam UMC dataset. Table 3 reports the mean and standard deviation of the classification results obtained from five independent runs using different random seeds. Results are reported across three evaluation sets: held-out public testset from PTB-XL and Chapman-Shaoxing datasets (Public), the held-out testset from Amsterdam UMC-weak dataset, and the expert-annotated Amsterdam UMC dataset (Amsterdam UMC Expert). Table 11 in the Appendix provides details on the classification of samples not belonging to the AF or SR class. For all metrics and evaluation sets, corresponding 95% bootstrap confidence intervals and effect sizes (Cliff’s *Δ*) are provided in the Appendix table 12.

Figure 4 displays precision and recall values across the three evaluation datasets for AF and SR classification. Additionally, results stratified by 1–4 and 5–8 available leads, by specific lead combination, and by individual lead ablation (see Appendix Tables 13, 14, 15, 16 and 17). To evaluate the repeatability of our findings, we further examined performance on the Amsterdam UMC Expert by comparing the original evaluation (first 5 s of each 10 s ECG) with a second labelled set constructed from the remaining 5 s. Mean results across seeds for both training configurations (with and without weak-Amsterdam UMC data) are summarised in Table 4, while the corresponding standard deviations are provided in the Appendix Table 18, detailed bootstrap confidence intervals and effect sizes for this comparison are again reported in the Appendix table 19. Figure 5 (with counts per subgroup given in Appendix Table 20) illustrates F1 scores stratified by patient sex and age group, again comparing models trained with and without weak-Amsterdam UMC data. Error bars represent 95% confidence intervals, and the underlying numerical results (including bootstrap confidence intervals and effect sizes for all subgroups) are available in the Appendix table 21. Finally, Appendix Tables 22 and 23 summarises model performance on this dataset stratified by clean versus noisy segments.

## Discussion

This study assesses whether an ECG-FM pre-trained on large, diverse datasets can robustly and generalisably detect AF and SR in ICU continuous monitoring. Our results show that ECG-FMs fine-tuned on carefully selected public diagnostic ECG datasets maintain strong performance when applied to expertly annotated ICU recordings obtained from continuous monitoring. Although annotation inconsistencies in public datasets can hinder performance, targeted fine-tuning substantially enhances clinical applicability. Adding weakly labelled in-house data further improved recall and overall F1 scores, with only modest reductions in precision (AF –0.012, SR –0.014; Table 3). These findings demonstrate that ECG-FMs, when fine-tuned appropriately, can effectively adapt to ICU arrhythmia detection tasks across diverse and unseen datasets.

Models trained exclusively on curated public datasets already generalised well to the Amsterdam UMC Expert test set (F1 - AF 0.814, SR 0.914; Table 3). Incorporating the Amsterdam UMC-weak data slightly improved these results (F1 - AF 0.830, SR 0.920; Table 3). After incorporating in-house data, the largest performance gains appeared on the Amsterdam UMC-weak test set, where F1 scores increased from 0.743 to 0.829 (AF) and from 0.957 to 0.970 (SR), indicating that institution-specific weak labels can enhance performance on noisy datasets. As expected, performance was highest on the public test set, where the model trained only on public data achieved F1 scores of 0.937 for AF and 0.977 for SR, and the model trained jointly with Amsterdam UMC-weak data achieved nearly identical results. The modest F1 decline from the public test set to the Amsterdam UMC Expert indicates strong generalisation. Performance was stable across temporal windows, with slightly greater–but still modest–variability across lead configurations, likely influenced by the imbalance between the 1–4 and 5–8 lead groups (Appendix Tables 13–14). The granular stratified analysis across all seven lead combinations confirms this pattern, with performance remaining broadly consistent (Appendix Table 16 ). An exploratory analysis stratified by signal quality (Appendix Tables 22 and 23) showed lower performance on noisy-but-interpretable segments compared with clean segments, although the relatively small number of noisy samples limits the strength of any conclusions. Demographic analyses showed minimal differences between sexes and greater variability across age groups for AF than for SR, again likely reflecting uneven and small subgroup sizes rather than genuine model bias (Figure 5) [35]. These results underline the need for cautious interpretation of subgroup comparisons in the presence of imbalanced sampling.

**Fig. 10:**
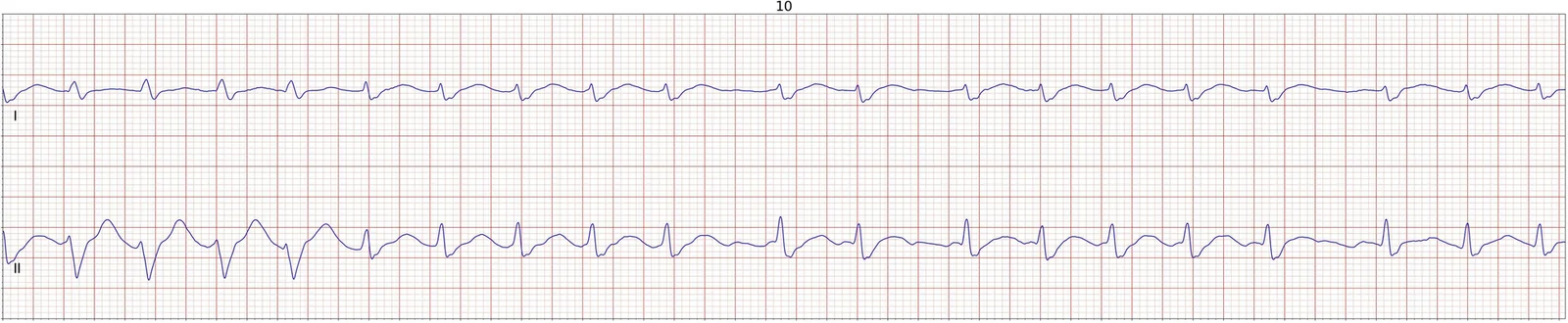
Predicted label: Other; Ground truth: AF

**Fig. 11:**
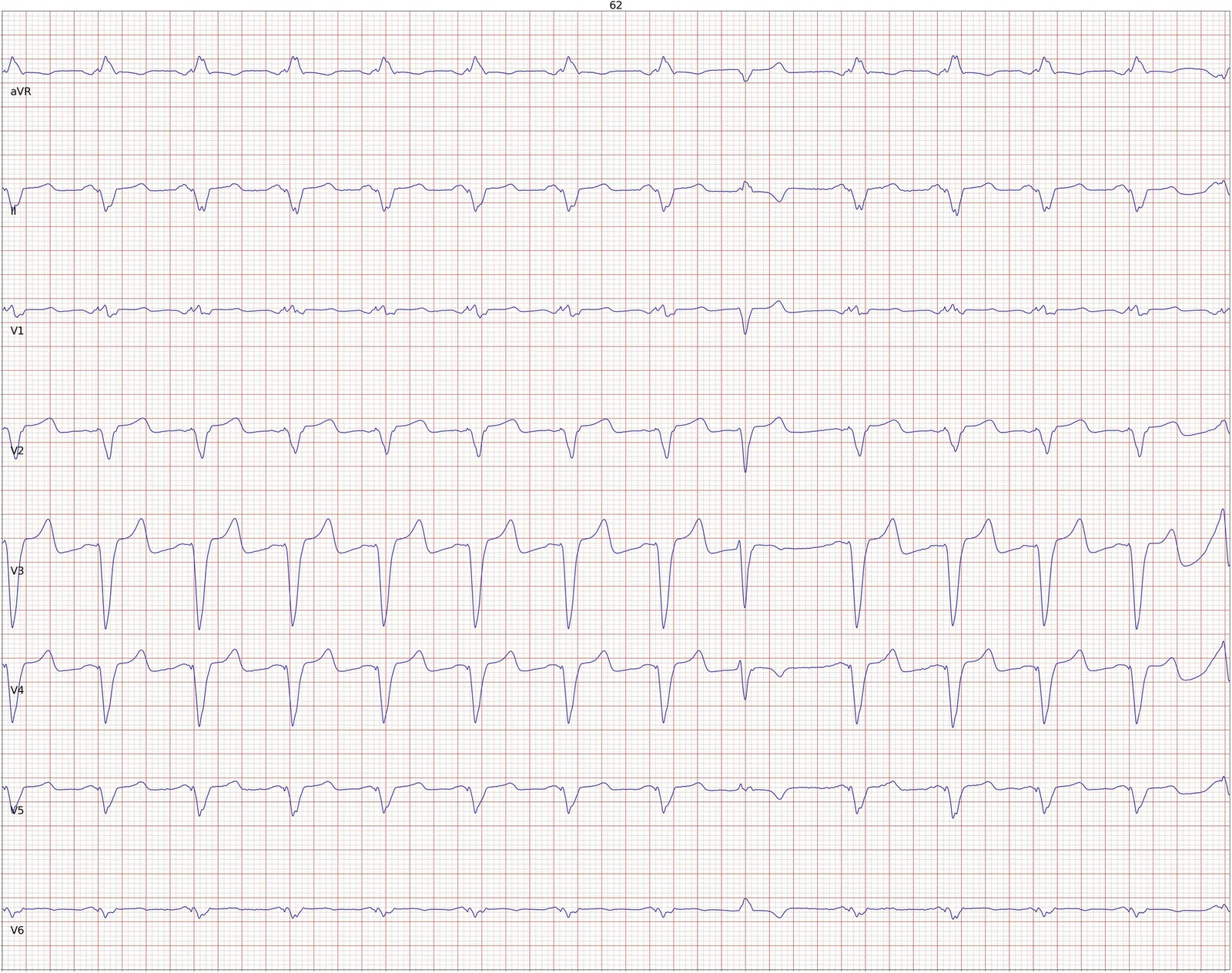
Predicted label: Other; Ground truth: SR

**Fig. 12:**
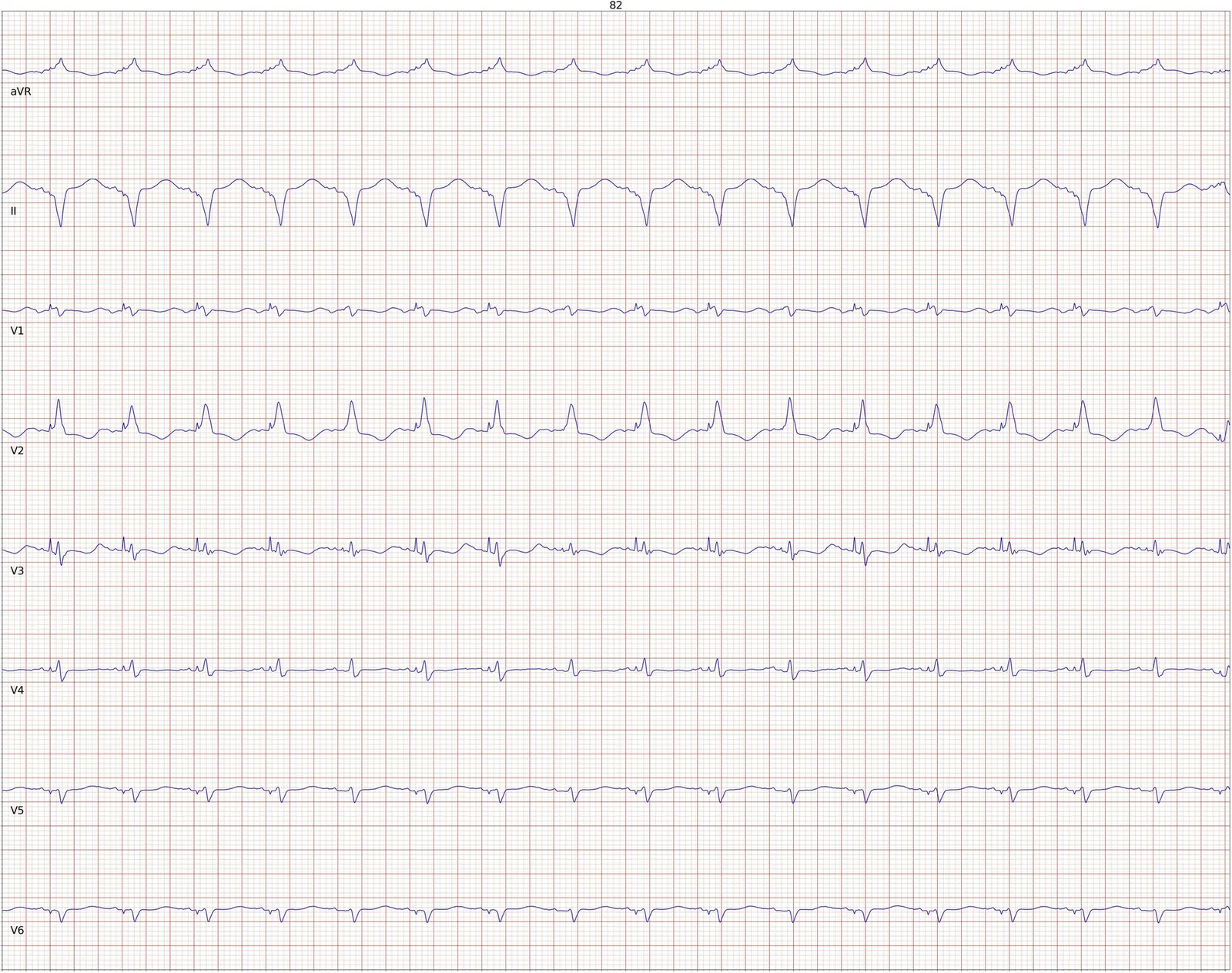
Predicted label: Other; Ground truth: SR

**Fig. 13:**
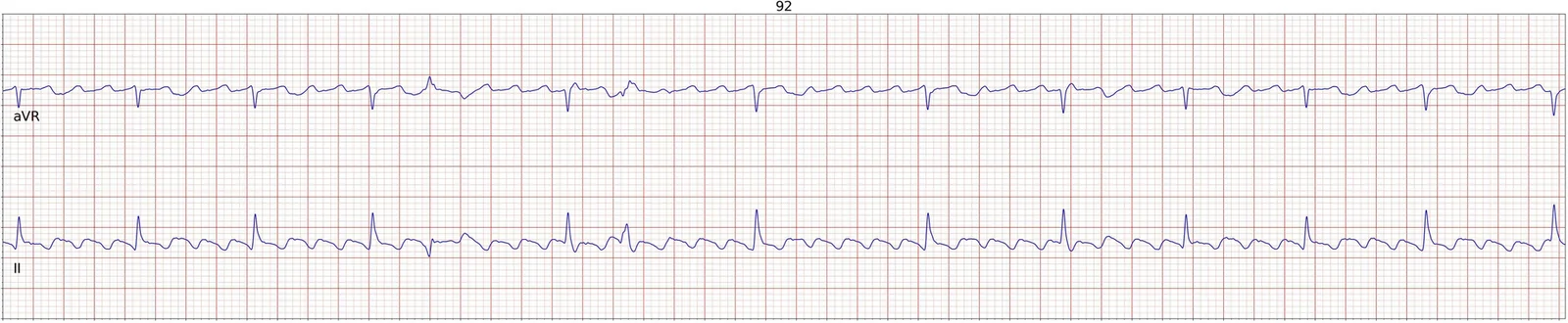
Predicted label: AF; Ground truth: Other

**Fig. 14:**
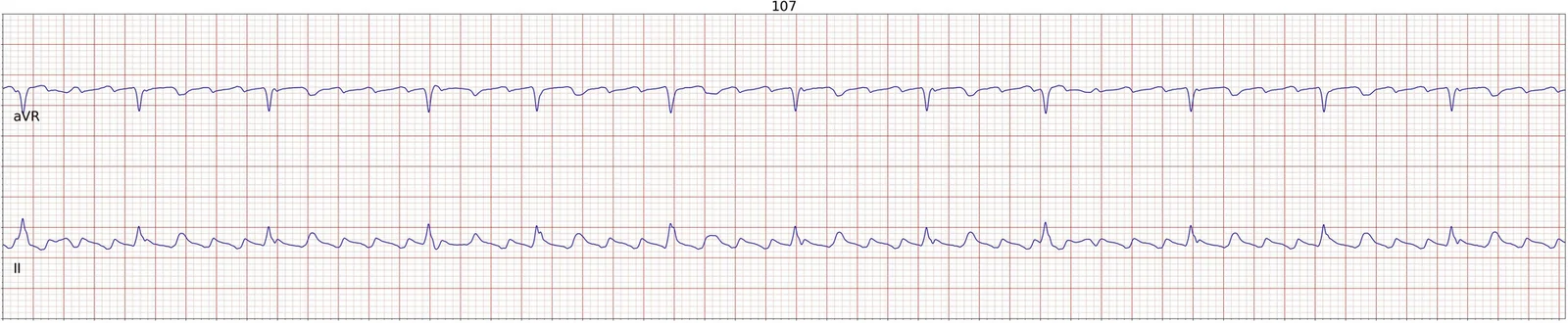
Predicted label: AF; Ground truth: Other

**Fig. 15:**
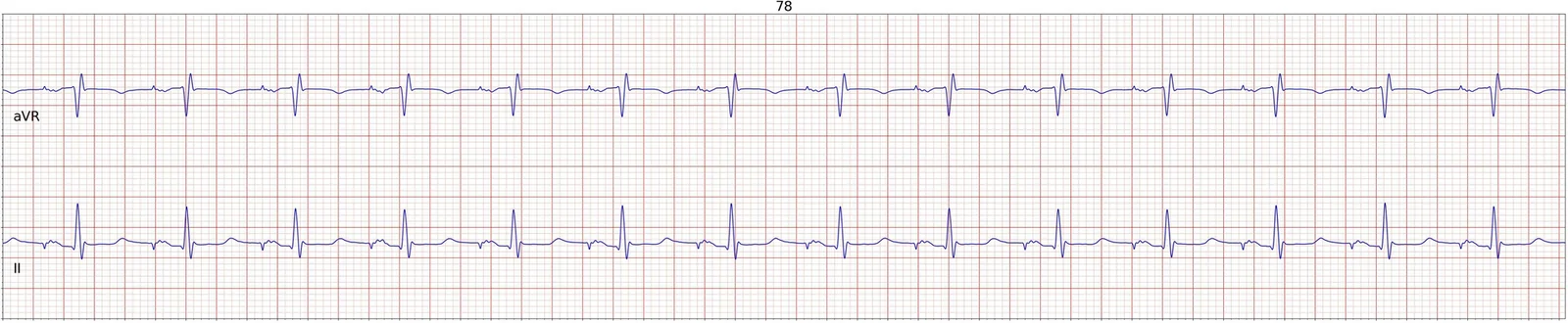
Predicted label: SR; Ground truth: Other

**Fig. 16:**
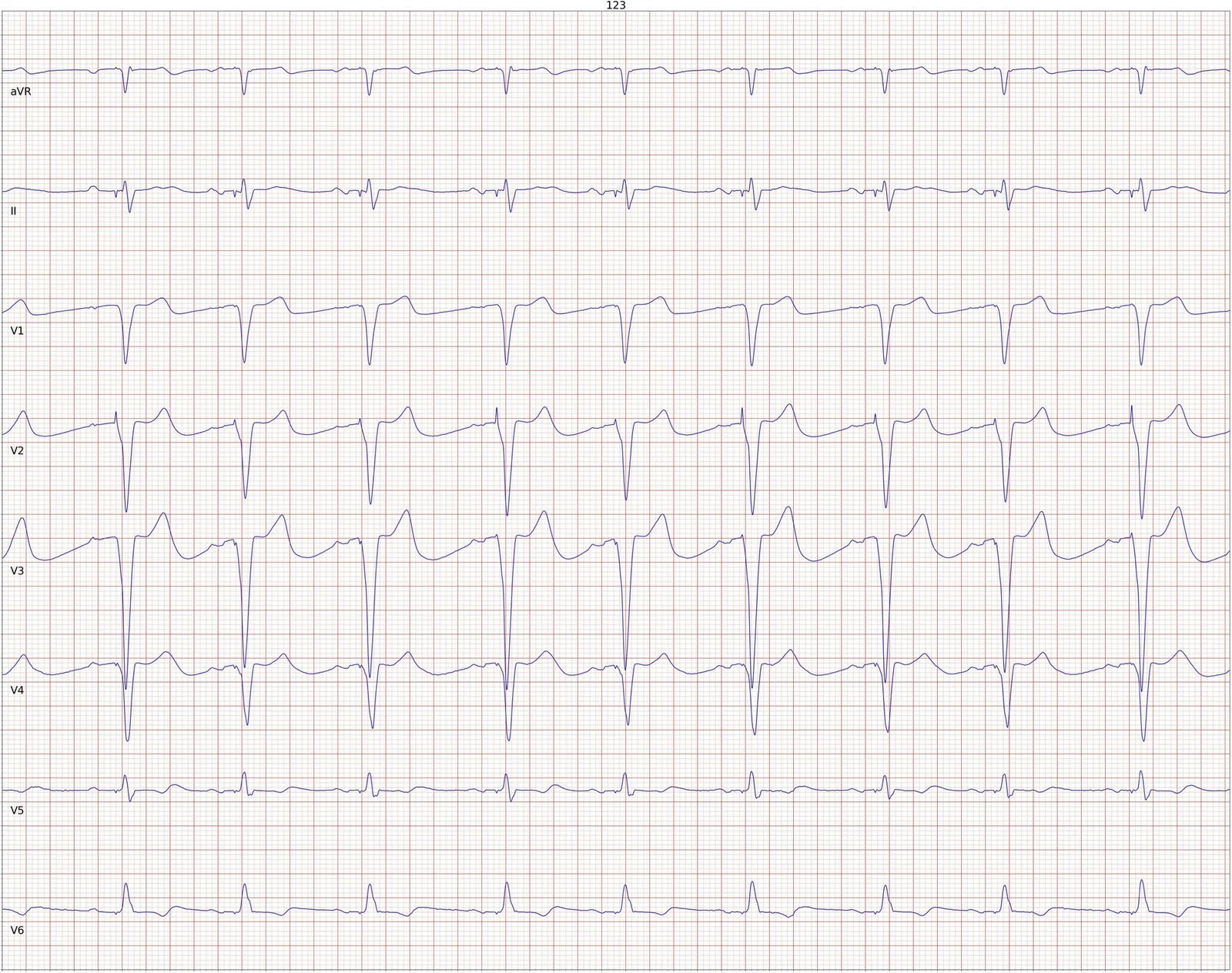
Predicted label: SR; Ground truth: Other

**Fig. 17:**
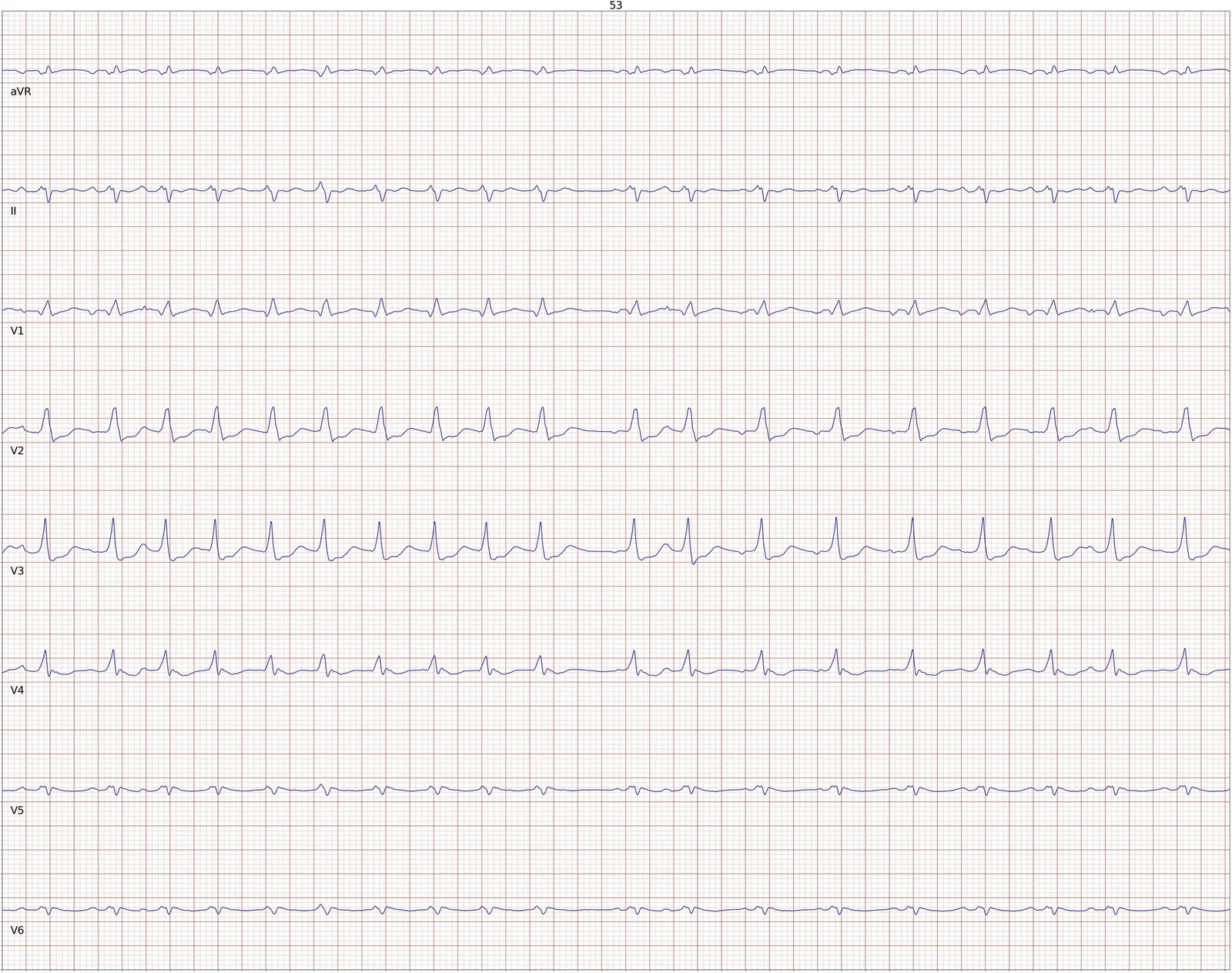
Predicted label: Other; Ground truth: AF

**Fig. 18:**
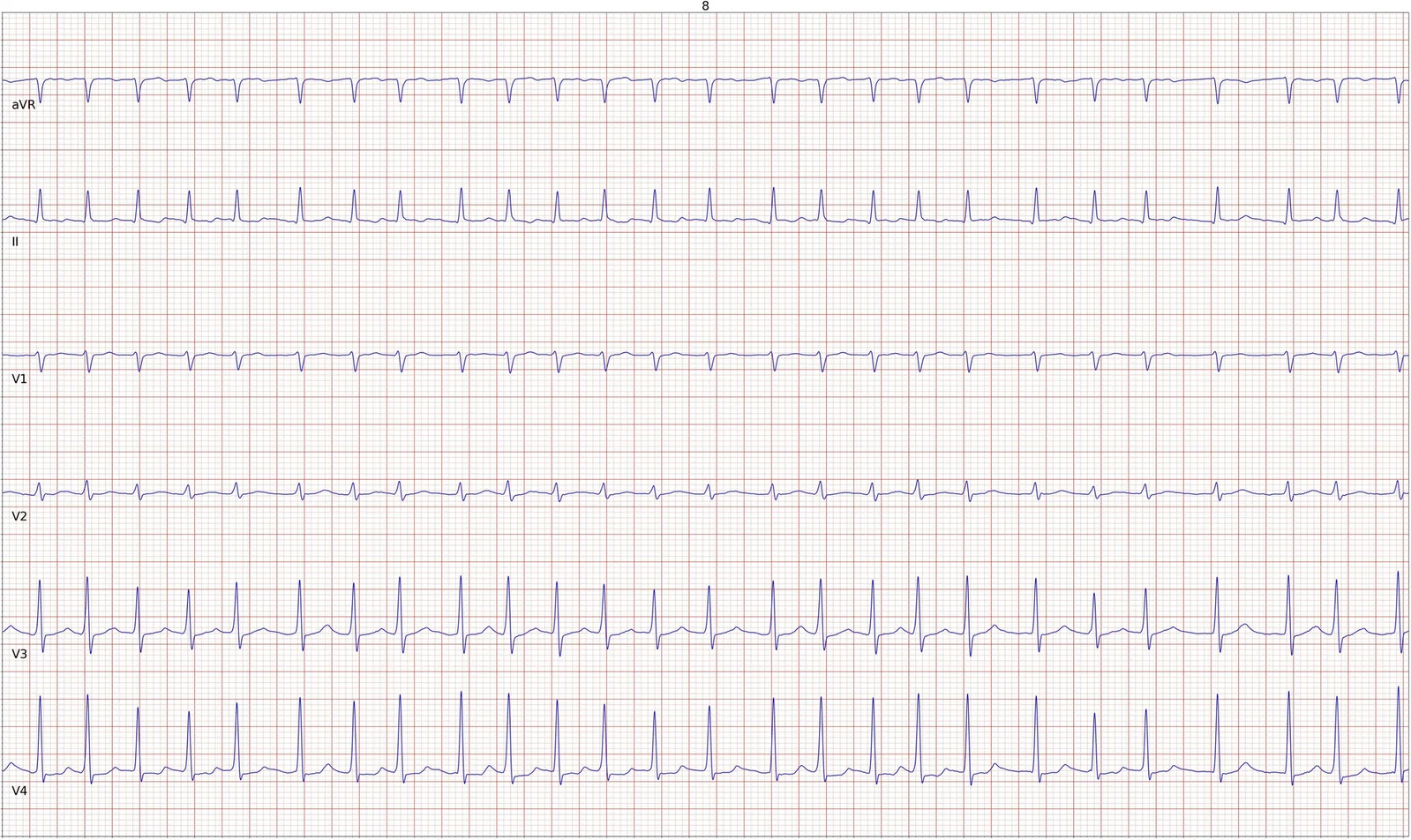
Predicted label: Other; Ground truth: AF

**Fig. 19:**
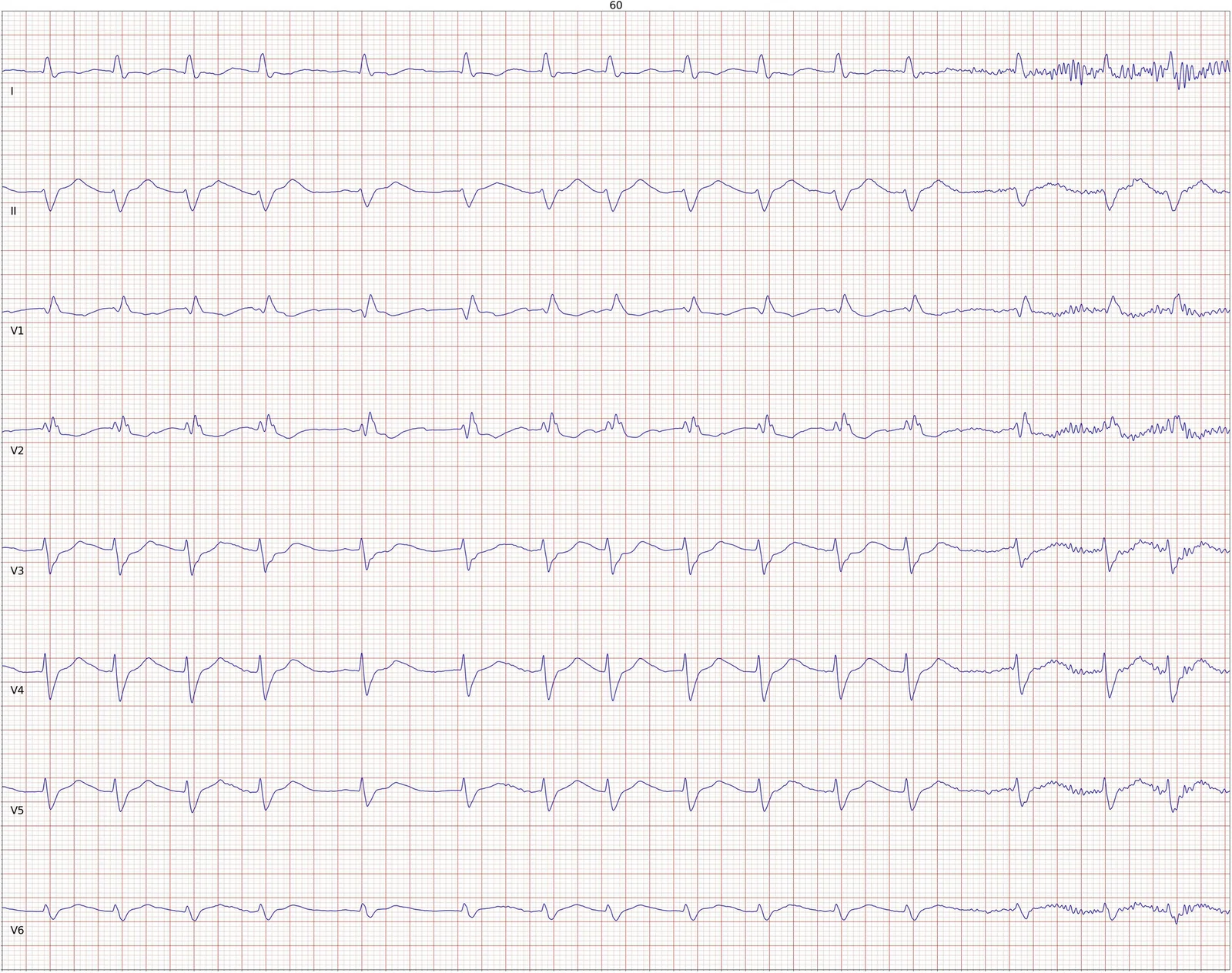
Predicted label: Other; Ground truth: AF

To better understand the model’s limitations, we examined a subset of samples that were misclassified in Experiment 3 (see Appendix, Figures 10 - 19 for representative examples). These error cases revealed several distinct patterns. In some instances, samples were inherently difficult to classify due to limited clinical information and the absence of a full 12-lead ECG. Our dataset comes from the ICU ward and includes recordings from postoperative cardiac patients with multiple pathologies. These cases are substantially more complex than the routine outpatient datasets typically used for training. Atrial flutter was occasionally misclassified as AF, and some pacemaker rhythms were interpreted as SR. We also noted cases in which the rhythm changed within the 10-second recording window; however, the model processed only the first 5 seconds, whereas the clinical annotation reflected the full 10 seconds. In addition, several cases were inherently challenging to classify, as continuous bedside monitoring in the ICU is associated with patient movement, ongoing medical interventions, and other artefacts that produce noisier and more complex ECG signals than those encountered in routine outpatient recordings. In some cases, the model’s predictions were clearly incorrect. One possible explanation could be that similar examples in the training data were mislabelled, causing the model to learn and reproduce those errors. These findings highlight the clinical complexity of the ICU population at the Amsterdam ICU and the challenges it poses.

Assessment of public datasets in Experiment 1 revealed considerable variation in label consistency. PTB-XL and Chapman-Shaoxing exhibited high F1 scores for AF, indicating strong internal label reliability and suitability for model fine-tuning. In contrast, Georgia and CPSC showed weaker and more variable performance, particularly for SR (Table 1), suggesting annotation inconsistencies or distribution shifts. Interestingly, the results of internal label validation align with the level of detail provided in each dataset’s documentation. Chapman-Shaoxing [24] and PTB-XL [22] offer the most robust transparency: the former employed a rigorous tri-level physician validation process, while the latter used a hybrid approach of automated interpretation followed by cardiologist review. The CPSC [33] dataset provides moderate detail, describing a semi-automated workflow that combined QRS detection with manual visual correction. In contrast, the Georgia dataset lacks formal procedural documentation entirely, providing only its geographical origin. These findings suggest that comprehensive documentation is a vital component of any dataset [36]. Conversely, a lack of transparency may serve as a subtle indicator of potential issues, e.g., labelling inconsistencies or underlying biases that warrant further investigation.

Evaluation of publicly released ECG-FM in Experiment 2 on the Amsterdam UMC dataset demonstrated limited generalisation. The MIMIC-IV-ECG fine-tuned model showed high AF recall (Table 2). These results underscore that label quality might be one of the crucial determinants of cross-institutional generalisation.

To contextualise our findings, we compared them with previous deep learning studies using well-annotated in-house ICU monitoring datasets. Both studies by Chen et al. [12, 13] merged AF and atrial flutter into a single class. The first study used weakly supervised learning to label ICU telemetry data and achieved F1 scores of 0.64–0.67. The later study classified SR versus the merged AF/atrial flutter class, excluding “other” rhythms such as pacemaker activity, and reported 84% sensitivity, 89% specificity, 55% precision, and 97% negative predictive value. While their recall is comparable to ours, their precision is notably lower. Moreover, unlike their simplified binary task, our models detect AF and SR within the full spectrum of rhythms encountered in continuous ICU monitoring, representing a substantially more challenging and clinically realistic scenario. It should be noted, however, that Chen et al. [13] evaluated on a larger test set of 869 segments compared to our 592. The performance disparity is likely driven by fundamental differences in model architecture and training scale. Both Chen et al. studies [12, 13] employed a Convolutional Neural Network (CNN) with an attention mechanism trained from scratch on approximately 10,000 ECG recordings, without any pretraining. ECG-FM, by contrast, is a large transformer-based foundation model that adopts a wav2vec 2.0–style design [37]: a convolutional feature extractor embeds raw ECG signals into latent representations, which are then passed to a BERT-Base–like Transformer [38] encoder that captures contextual dependencies across the full signal – enabling the model to jointly model both local waveform morphology and long-range temporal structure. Critically, ECG-FM was pretrained on approximately 1.5 million 12-lead ECG segments, roughly 150 times more data than used by Chen et al., enabling far richer and more generalisable representations prior to any task-specific fine-tuning.

Overall, these results suggest that foundation models like ECG-FM can achieve robust and generalizable AF and SR detection in the ICU when fine-tuned on carefully selected public datasets and supplemented with institution-specific weakly labelled data. This approach provides a pragmatic pathway to developing scalable, high-performance ECG models that are adaptable to diverse clinical environments.

Regarding clinical deployment, the intended use case is real-time rhythm monitoring support, where model outputs would assist ICU nurses in validating monitor-detected arrhythmias, reducing manual review burden and improving documentation reliability. Alarm generation represents another potential application; however, determining the appropriate precision-recall operating point would require dedicated discussion with ICU nurses, clinical staff, and relevant stakeholders, as well as prospective studies to establish whether missed detections or false alarms are more undesirable in the target deployment context. Furthermore, the model’s robustness to reduced lead configurations suggests potential utility in reducing reliance on full 12-lead ECGs for rhythm confirmation, which currently adds to nursing workload. Finally, this work represents a natural first step towards predictive rhythm monitoring, where sufficiently reliable detection could enable the construction of larger annotated datasets and ultimately support proactive rather than reactive clinical intervention.

While our analysis provides important insights into ECG-FM data, it is important to acknowledge certain limitations. The first is that dataset consistency was assessed using a single ECG-FM. It is possible that the way this model learns and represents ECG features may not fully capture label inconsistencies in all datasets, and therefore, the observed performance differences could, in part, reflect model-specific biases rather than purely annotation quality. However, because ECG-FM was pre-trained on the PhysioNet 2021 Challenge datasets themselves, it provides a reasonable and relevant proxy for evaluating internal label reliability in this specific context. Furthermore, in Experiment 1, labelling consistency was utilised as a proxy for label quality. However, it is important to note that high internal validation accuracy does not always equate to clinical accuracy; a model may achieve high consistency by simply learning systematic, machine-generated errors present in the source data. Consequently, labels may appear reliable within the model’s framework while remaining fundamentally incorrect from a medical standpoint. Additionally, the applied filtering and model inference both operate on 5-second windows, which introduces an inherent delay that should be considered when translating the approach to a strictly real-time bedside deployment.

Beyond model-related considerations, several data-related limitations should be acknowledged. First, all Amsterdam UMC ECGs contained only a subset of the measured leads, and we are still investigating the underlying cause of this. Second, the relatively small size of the expert-annotated Amsterdam UMC dataset remains a constraint. Although 600 ECG segments from 226 patients were sampled in a stratified manner to preserve the distribution of 16 rhythm categories observed in clinical practice, the dataset’s limited scale restricts the statistical power of subgroup analyses and may not capture the full diversity of ICU populations. This issue is compounded by uneven sample sizes between males and females and across age categories, which could influence the observed demographic effects and warrants further investigation in larger, balanced cohorts. Third, the temporal alignment of nurse-derived weak labels introduces a potential source of label noise. Nursing documentation timestamps in Epic are frequently rounded to the nearest full hour, meaning the recorded time may deviate from the true observation time. Combined with the possibility of delayed or retrospective documentation, this introduces temporal uncertainty that cannot be fully resolved at the matching stage. We therefore treat these labels as weak supervision and note this as an inherent limitation of the dataset construction. Finally, as all data were obtained from a single institution (Amsterdam UMC), site-specific factors, such as patient demographics, monitoring equipment, data storage and warehousing procedures, and annotation practices, may have influenced the results. Future work should therefore aim to extend both the size and institutional diversity of expert-labelled datasets to enable more comprehensive and generalisable evaluations of ECG-FMs across different clinical environments. To address these limitations, potential next steps include multi-centre data collection, the use of federated validation and training frameworks, and harmonisation of lead configurations across sites. As a small initial step towards another institution validation, we also conducted a preliminary external evaluation on continuous ICU ECG from the MIMIC Waveform Database (Appendix Table 24) [39, 40], although limited in size and based on single-annotator labels, this analysis provides early support for the transferability of our approach. In addition, dedicated experiments on a broader set of ICU rhythm categories (e.g., atrial flutter and other arrhythmias) are needed to more fully characterise and evaluate model behaviour beyond AF and SR.

## Conclusion

In this study, we evaluated ECG-FMs for detecting AF and SR in the ICU using a multi-stage experimental design. Most importantly, our results demonstrate that ECG-FMs fine-tuned on carefully selected public datasets can maintain strong generalisation to external, expertly annotated ICU data coming from continuous monitoring. Incorporating weakly labelled in-house ICU data further enhanced recall and overall F1 scores, though at a modest cost to precision. These findings highlight that ECG-FMs can adapt effectively to diverse clinical data distributions and heterogeneous ECG lead configurations, offering a promising and scalable approach for arrhythmia detection in critical care settings.

## Figures and Tables

**Fig. 6:**
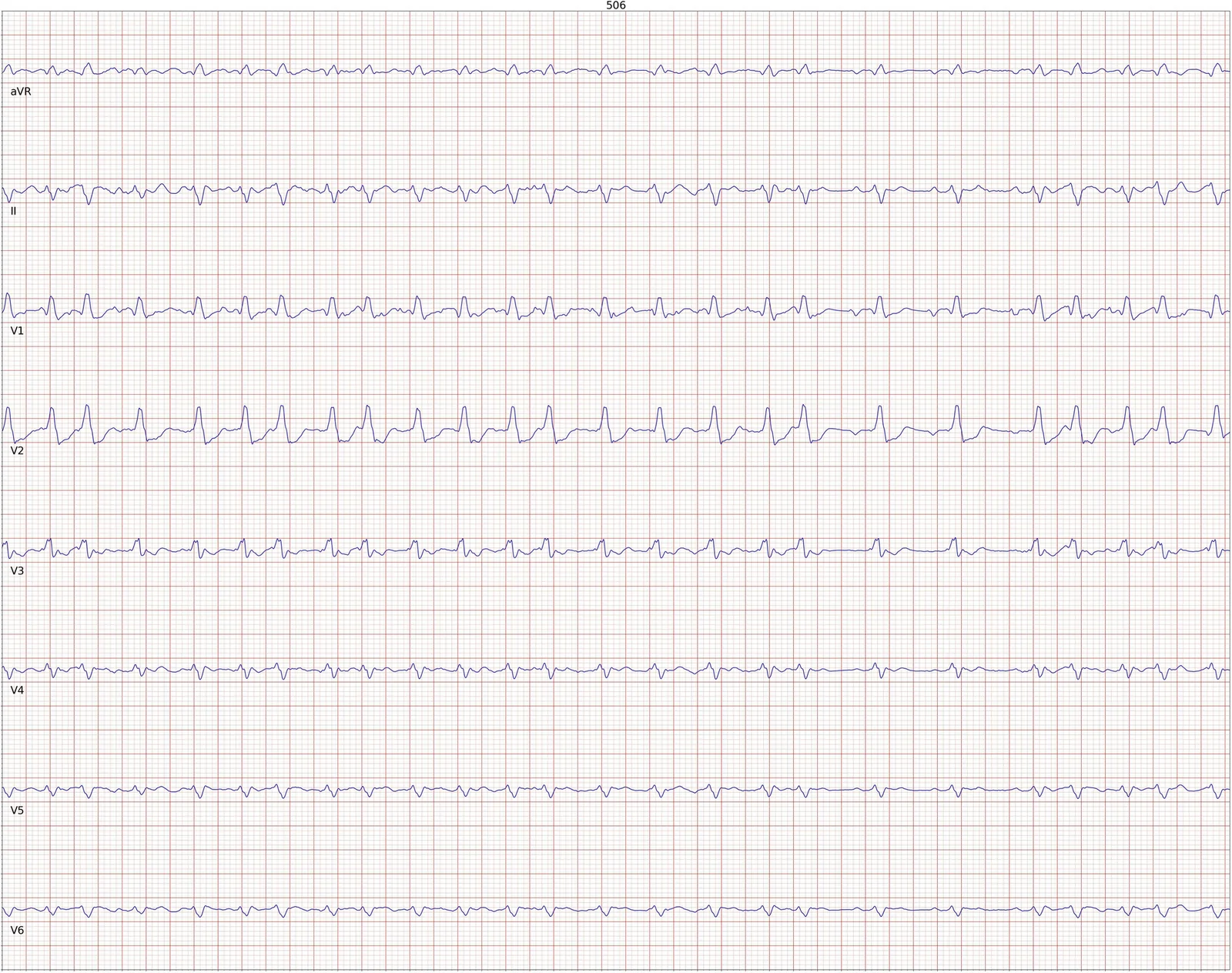
Sample annotated as both AF and SR with the following annotator comment "AF with three sinus beats"

**Fig. 7:**
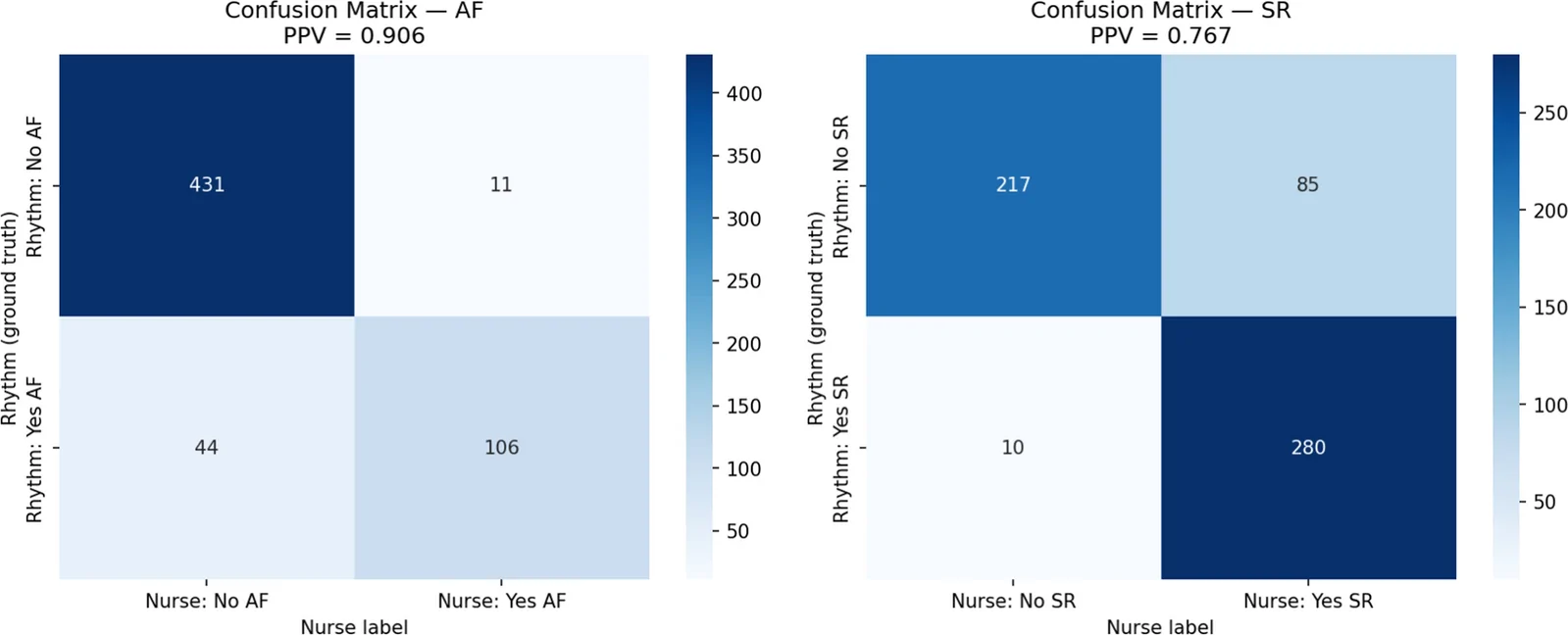
Confusion matrices along with PPV comparing nurse-assigned labels (AF and SR) against clinician-annotated rhythm labels in Amsterdam UMC Expert Dataset

**Fig. 8:**
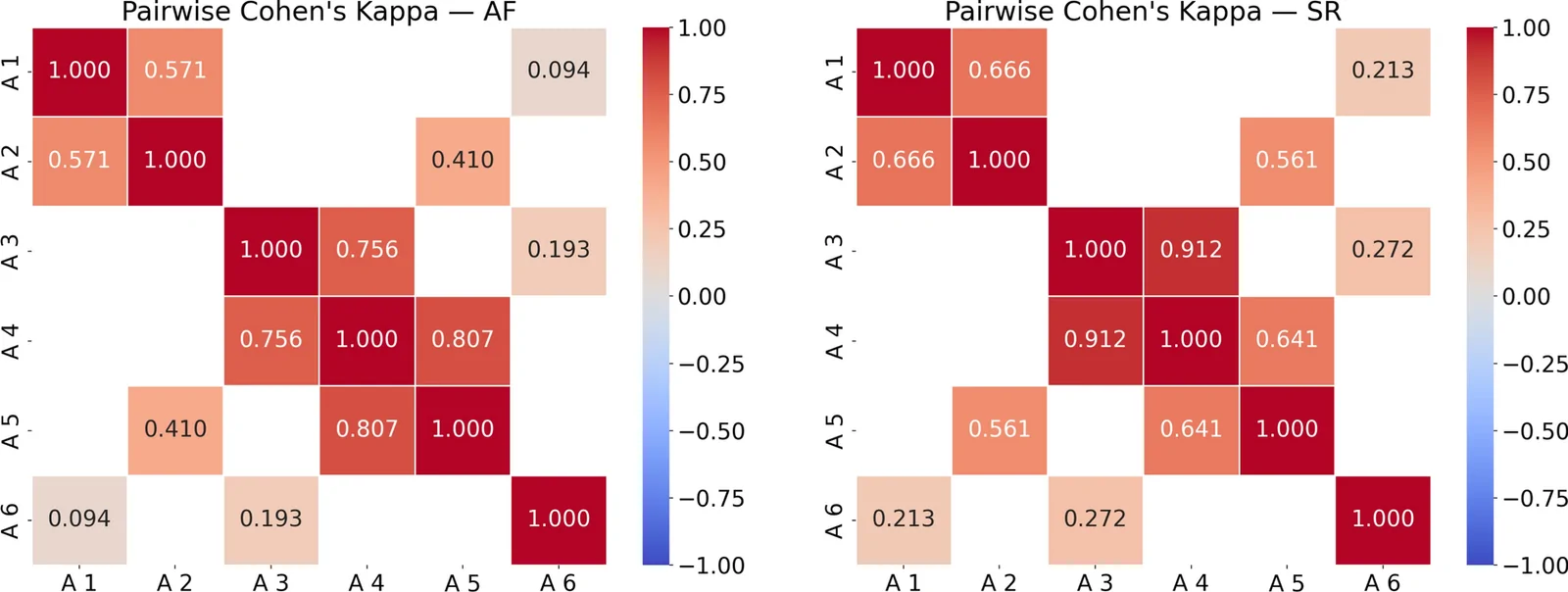
Pairwise Cohen’s Kappa among junior annotators for AF (left) and SR (right). Cells show kappa values for each annotator pair; empty cells indicate no shared samples

**Fig. 9:**
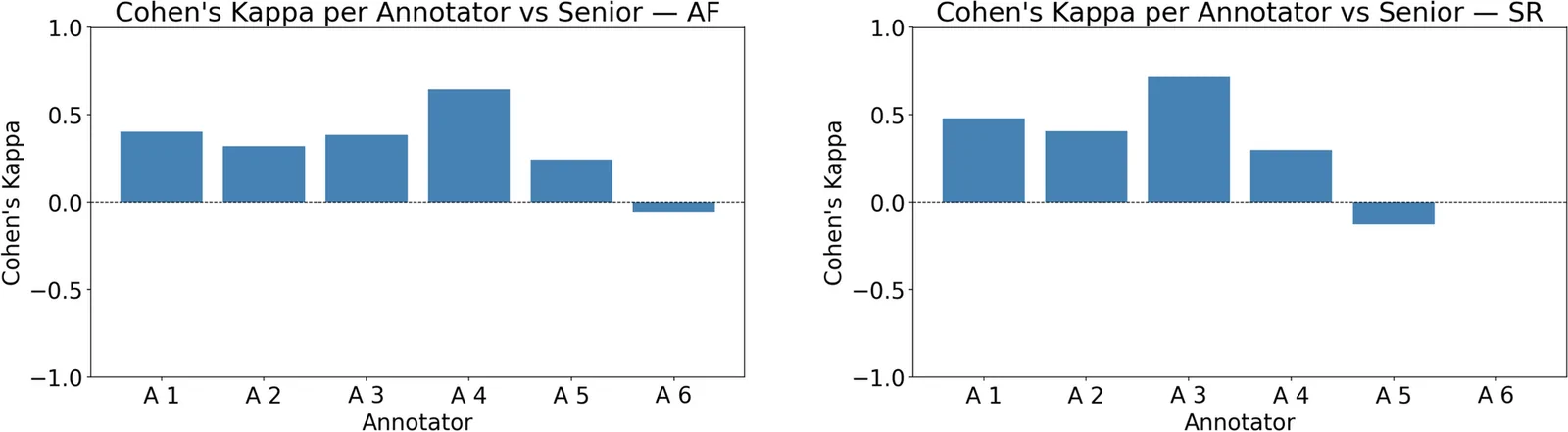
Cohen’s Kappa between each junior annotator and the senior annotator for AF (left) and SR (right). Only samples where the senior annotator provided a label are included

## Data Availability

The Physionet datasets used in this study are publicly available from the official PhysioNet *Will Two Do? Varying Dimensions in Electrocardiography: The PhysioNet/Computing in Cardiology Challenge 2021* repository [5]. The repository is accessible at https://physionet.org/content/challenge-2021/1.0.3/. The internal Amsterdam UMC datasets analysed in this study cannot be made publicly available in order to protect patient privacy.

## Appendix A

### A.1 Datasets characteristics

See Table 5 and 6.

**Table 5:** Segment counts per dataset: AF, SR, AF+SR (segments labelled with both classes simultaneously), Other (segments labelled as neither AF nor SR)

**Dataset**	**Total**	**AF**	**SR**	**AF+SR**	**Other**
*Public datasets*					
PTBXL	21,837	1,514	18,966	37	1,394
CPSC2018	6,877	1,221	918	0	4,738
Georgia	10,344	570	4,690	29	5,113
ChapmanShaoxing	10,247	1,780	7,283	0	1,184
*Amsterdam UMC datasets*					
Weakly labelled	8,580	874	7,325	0	381
Expert-annotated	592	117	365	1	111

**Table 6:** Train/val/test split sizes for the public datasets (PTBXL + ChapmanShaoxing) and the Amsterdam UMC weakly labelled dataset

**Dataset**	**Train**			**Val**			**Test**		
	**n**	**AF**	**SR**	**n**	**AF**	**SR**	**n**	**AF**	**SR**
*Public (PTBXL + ChapmanShaoxing)*									
	22,458	2,306	18,368	3,272	336	2,693	6,354	652	5,188
*Amsterdam UMC-weak*									
	6,006	612	5,124	1,287	131	1,102	1,287	131	1,099

*n* = total segments; AF and SR columns show positive-case counts

### A.2 The amsterdam UMC expert dataset characteristics

See Fig. 6, 7, 8, and 9, and Table 7 and 8.

**Table 7:** Detailed nurse-assigned rhythm labels for samples whose ground truth is neither AF nor SR (*n = 111*) in Amsterdam UMC Expert Dataset

**Rhythm**	**Count**	**Percentage**
Pacemaker rhythm	46	41.4%
Atrial fibrillation	24	21.6%
Atrial flutter	13	11.7%
AV-junctional rhythm	9	8.1%
Ventricular rhythm	8	7.2%
Sinus rhythm	6	5.4%
Atrial rhythm	2	1.8%
Ventricular fibrillation	1	0.9%
Asystole	1	0.9%
Ventricular tachycardia	1	0.9%
**Total**	111	100%

**Table 8:** Cohen’s Kappa between each annotator and the senior annotator for SR and AF

**Annotator**	**AF**	**SR**
A 1	0.404	0.479
A 2	0.319	0.407
A 3	0.383	0.716
A 4	0.644	0.299
A 5	0.244	-0.127
A 6	-0.054	0.002
**Median**	**0.353**	**0.351**

See Table 9 and 10

**Table 9:** Five-fold cross-validation performance on the two Amsterdam UMC datasets using the ECG-FM representation with a linear classifier

**Class**	**Metric**	**Amsterdam UMC Expert**	**Amsterdam UMC-weak**
AF	Accuracy	*0.929± 0.028*	*0.960± 0.012*
	Precision	*0.866± 0.122*	*0.821± 0.087*
	Recall	*0.786± 0.128*	*0.794± 0.033*
	F1	*0.814± 0.074*	*0.805± 0.046*
SR	Accuracy	*0.877± 0.027*	*0.930± 0.009*
	Precision	*0.902± 0.062*	*0.957± 0.010*
	Recall	*0.905± 0.047*	*0.961± 0.016*
	F1	*0.901± 0.016*	*0.959± 0.006*

Values are mean ± std across five folds

**Table 10:** Fold segment counts for the five-fold cross-validation on the Amsterdam UMC Expert and Amsterdam UMC-weak datasets

**Dataset**	**Fold**	**Total**	**AF**	**SR**
Amsterdam UMC Expert	1	119	24	71
	2	119	24	73
	3	118	23	83
	4	118	23	68
	5	118	23	70
	*Total*	592	117	365
Amsterdam UMC-weak	1	1715	174	1477
	2	1715	175	1455
	3	1715	175	1466
	4	1715	175	1452
	5	1715	175	1470
	*Total*	8575	874	7320

### A.4 Additional results experiment 3

See Table 11, 12, 13, 14, 15, 16, 17, 18, 19, 20, 21 and 23.

**Table 11:** Classification of “Other” samples (AF*=0*, SR*=0*) across the Amsterdam UMC Expert test set and model variants

Variant	*n* Other	AF only	SR only	Both AF+SR	Neither
Pub	111	*10.8± 2.9*	*26.2± 3.6*	*0.0± 0.0*	*74.0± 3.9*
Pub+Amsterdam UMC-weak	111	*11.2± 4.3*	*27.6± 2.3*	*0.6± 0.9*	*71.6± 5.3*

Values are mean ± and std sample counts across five random seeds. Neither = no class exceeded the decision threshold (implicit abstention)

**Table 12:** Detailed statistical comparison of Pub vs. Pub+Amsterdam UMC-weak training regimes

Class	Metric	**Pub**		**Pub+ UMC-weak**		*Δ*	Ovlp	Cliff
		Mean±Std	95% CI	Mean±Std	95% CI			
**Public test set**								
AF	Acc	*0.990± 0.001*	[0.987, 0.993]	*0.988± 0.001*	[0.984, 0.991]	*-0.002*	*+0.004*	*+1.00*
AF	Prec	*0.948± 0.012*	[0.922, 0.975]	*0.919± 0.017*	[0.881, 0.949]	*-0.029*	*+0.027*	*+0.92*
AF	Rec	*0.956± 0.015*	[0.920, 0.977]	*0.971± 0.008*	[0.951, 0.988]	*+0.015*	*+0.026*	*-0.72*
AF	F1	*0.952± 0.003*	[0.938, 0.964]	*0.944± 0.005*	[0.927, 0.959]	*-0.008*	*+0.021*	*+0.92*
SR	Acc	*0.964± 0.001*	[0.960, 0.969]	*0.964± 0.000*	[0.959, 0.969]	*-0.001*	*+0.009*	*+0.24*
SR	Prec	*0.962± 0.002*	[0.956, 0.968]	*0.963± 0.001*	[0.957, 0.968]	*+0.001*	*+0.011*	*-0.28*
SR	Rec	*0.996± 0.001*	[0.994, 0.998]	*0.995± 0.001*	[0.991, 0.997]	*-0.001*	*+0.003*	*+0.76*
SR	F1	*0.979± 0.001*	[0.976, 0.982]	*0.979± 0.000*	[0.976, 0.981]	*+0.000*	*+0.005*	*+0.36*
**Amsterdam UMC Weak test set**								
AF	Acc	*0.945± 0.004*	[0.931, 0.959]	*0.964± 0.005*	[0.950, 0.976]	*+0.019*	*+0.009*	*-1.00*
AF	Prec	*0.715± 0.042*	[0.619, 0.824]	*0.805± 0.054*	[0.693, 0.906]	*+0.090*	*+0.131*	*-0.76*
AF	Rec	*0.779± 0.058*	[0.648, 0.882]	*0.858± 0.040*	[0.769, 0.944]	*+0.079*	*+0.113*	*-0.80*
AF	F1	*0.743± 0.017*	[0.674, 0.805]	*0.829± 0.012*	[0.773, 0.879]	*+0.086*	*+0.032*	*-1.00*
SR	Acc	*0.928± 0.003*	[0.913, 0.943]	*0.948± 0.004*	[0.934, 0.961]	*+0.020*	*+0.009*	*-1.00*
SR	Prec	*0.972± 0.004*	[0.959, 0.983]	*0.965± 0.006*	[0.949, 0.979]	*-0.007*	*+0.020*	*+0.60*
SR	Rec	*0.944± 0.006*	[0.927, 0.960]	*0.975± 0.008*	[0.954, 0.987]	*+0.031*	*+0.006*	*-1.00*
SR	F1	*0.957± 0.002*	[0.948, 0.966]	*0.970± 0.002*	[0.961, 0.977]	*+0.013*	*+0.005*	*-1.00*
**Amsterdam UMC Expert test set**								
AF	Acc	*0.931± 0.006*	[0.907, 0.953]	*0.935± 0.006*	[0.911, 0.956]	*+0.004*	*+0.042*	*-0.36*
AF	Prec	*0.867± 0.026*	[0.785, 0.941]	*0.855± 0.045*	[0.742, 0.939]	*-0.012*	*+0.154*	*+0.04*
AF	Rec	*0.769± 0.052*	[0.649, 0.875]	*0.809± 0.025*	[0.722, 0.889]	*+0.040*	*+0.153*	*-0.44*
AF	F1	*0.814± 0.022*	[0.742, 0.877]	*0.830± 0.009*	[0.771, 0.882]	*+0.016*	*+0.106*	*-0.44*
SR	Acc	*0.894± 0.005*	[0.867, 0.919]	*0.899± 0.003*	[0.873, 0.924]	*+0.005*	*+0.046*	*-0.68*
SR	Prec	*0.917± 0.010*	[0.883, 0.950]	*0.903± 0.008*	[0.870, 0.935]	*-0.014*	*+0.052*	*+0.76*
SR	Rec	*0.911± 0.012*	[0.875, 0.946]	*0.938± 0.010*	[0.905, 0.965]	*+0.027*	*+0.041*	*-0.92*
SR	F1	*0.914± 0.005*	[0.890, 0.935]	*0.920± 0.003*	[0.898, 0.940]	*+0.006*	*+0.037*	*-0.92*

Mean and std computed across five random seeds. Bootstrap 95% CIs resampled over test patients (10,000 resamples, pooled across seeds).*Δ*= mean difference (Pub+Amsterdam UMC-weak minus Pub). Ovlp = CI overlap: positive values indicate the two CIs share a common region (models not clearly distinguishable); negative values indicate a gap between the CIs. Cliff = Cliff’s*Δ*; negative values indicate Pub+Amsterdam UMC-weak performs better;$|\text {Cliff}| \ge 0.474$denotes a large effect size.$^{\dagger }$Entries where CIs do not overlap (Ovlp*<0*) and the effect size is large$((|\text {Cliff}| \ge 0.474))$, indicating a clearly distinguishable, large effect favouring one of the training regimes

**Table 13:** Experiment 3 results: Classification performance of two fine-tuned ECG-FMs - one trained on public data only (Pub) and the other on public plus Amsterdam UMC weakly labelled data (Pub+Amsterdam UMC-weak) on Amsterdam UMC Expert test set

**Training setup**	**Class**	**Accuracy**	**Precision**	**Recall**	**F1**
**Pub**					
1–4 leads	AF	0.946±0.006	0.846±0.039	0.819±0.060	0.830±0.022
	SR	0.918±0.003	0.940±0.008	0.936±0.012	0.938±0.003
5-8 leads	AF	0.919±0.007	0.881±0.018	0.741±0.050	0.804±0.024
	SR	0.876±0.010	0.897±0.015	0.888±0.014	0.892±0.008
**Pub + Amsterdam UMC-weak**					
1–4 leads	AF	0.946±0.008	0.850±0.056	0.814±0.020	0.831±0.019
	SR	0.919±0.008	0.915±0.009	0.969±0.007	0.941±0.006
5–8 leads	AF	0.925±0.006	0.895±0.042	0.805±0.031	0.830±0.007
	SR	0.884±0.003	0.892±0.014	0.909±0.014	0.900±0.002

**Table 14:** Number of AF, SR and other samples in Amsterdam UMC Expert test set grouped by different valid lead groups

**Lead Group**	**AF Samples**	**SR Samples**	**Other Samples**	**Total**
1–4 leads	42	173	45	260
5–8 leads	75	192	65	332

Results are reported separately for recordings with 1–4 available leads (grouped together) and recordings with 5–8 available leads (grouped together). Performance metrics are averaged across random seeds, with the standard deviation indicated after ‘±’

**Table 15:** Lead combination counts in the Amsterdam UMC expert-annotated test set

Lead combination	*n* leads	Total	AF	SR
(II, aVR, V1, V2, V3, V4, V5, V6)	8	279	62	168
(II, aVR)	2	201	28	138
(II, aVR, V1)	3	22	6	16
(II)	1	20	3	11
(I, II, V1, V2, V3, V4, V5, V6)	8	19	6	4
(I, II)	2	11	4	5
Other	7	40	8	23
Total		592	117	365

Lead combinations with fewer than 10 segments are pooled as *Other*. AF and SR counts reflect true labels (multi-label; a segment can carry both)

**Table 16:** Model performance on the Amsterdam UMC expert-annotated test set stratified by lead combination

**Class**	**Metric**	**(II, aVR, V1–V6)**		**(II, aVR)**	
		**Pub**	**Pub+Weak**	**Pub**	**Pub+Weak**
AF	Accuracy	*0.927± 0.005*	*0.928± 0.003*	*0.960± 0.005*	*0.964± 0.011*
	Precision	*0.901± 0.020*	*0.875± 0.035*	*0.856± 0.043*	*0.894± 0.075*
	Recall	*0.755± 0.040*	*0.794± 0.039*	*0.864± 0.064*	*0.850± 0.016*
	F1	*0.821± 0.019*	*0.831± 0.006*	*0.858± 0.020*	*0.870± 0.033*
SR	Accuracy	*0.868± 0.010*	*0.880± 0.005*	*0.934± 0.005*	*0.937± 0.008*
	Precision	*0.896± 0.014*	*0.894± 0.015*	*0.945± 0.007*	*0.921± 0.008*
	Recall	*0.883± 0.016*	*0.908± 0.016*	*0.961± 0.011*	*0.994± 0.003*
	F1	*0.890± 0.008*	*0.901± 0.004*	*0.953± 0.004*	*0.956± 0.005*

**Class**	**Metric**	**(II, aVR, V1)**		**(II)**	
		**Pub**	**Pub+Weak**	**Pub**	**Pub+Weak**
AF	Accuracy	*0.909± 0.000*	*0.918± 0.020*	*0.900± 0.035*	*0.850± 0.035*
	Precision	*0.767± 0.037*	*0.771± 0.048*	*0.833± 0.236*	*0.567± 0.253*
	Recall	*0.967± 0.075*	*1.000± 0.000*	*0.467± 0.183*	*0.333± 0.000*
	F1	*0.852± 0.011*	*0.870± 0.029*	*0.573± 0.159*	*0.407± 0.060*
SR	Accuracy	*0.800± 0.041*	*0.836± 0.041*	*0.910± 0.042*	*0.920± 0.027*
	Precision	*1.000± 0.000*	*1.000± 0.000*	*0.872± 0.041*	*0.874± 0.039*
	Recall	*0.725± 0.056*	*0.775± 0.056*	*0.982± 0.041*	*1.000± 0.000*
	F1	*0.840± 0.039*	*0.872± 0.036*	*0.923± 0.036*	*0.933± 0.022*

**Class**	**Metric**	**(I, II, V1–V6)**		**(I, II)**	
		**Pub**	**Pub+Weak**	**Pub**	**Pub+Weak**
AF	Accuracy	*0.832± 0.024*	*0.832± 0.058*	*0.818± 0.000*	*0.855± 0.050*
	Precision	*0.756± 0.062*	*0.744± 0.106*	*1.000± 0.000*	*1.000± 0.000*
	Recall	*0.700± 0.075*	*0.733± 0.091*	*0.500± 0.000*	*0.600± 0.137*
	F1	*0.724± 0.037*	*0.734± 0.079*	*0.667± 0.000*	*0.743± 0.104*
SR	Accuracy	*0.905± 0.024*	*0.853± 0.024*	*0.909± 0.000*	*0.800± 0.041*
	Precision	*0.800± 0.112*	*0.630± 0.067*	*0.833± 0.000*	*0.696± 0.040*
	Recall	*0.750± 0.000*	*0.750± 0.000*	*1.000± 0.000*	*1.000± 0.000*
	F1	*0.771± 0.048*	*0.683± 0.037*	*0.909± 0.000*	*0.821± 0.029*

**Class**	**Metric**	**Other**			
		**Pub**	**Pub+Weak**		
AF	Accuracy	*0.915± 0.029*	*0.950± 0.035*		
	Precision	*0.845± 0.029*	*0.819± 0.096*		
	Recall	*0.700± 0.143*	*0.975± 0.056*		
	F1	*0.761± 0.099*	*0.889± 0.075*		
SR	Accuracy	*0.910± 0.014*	*0.920± 0.011*		
	Precision	*0.930± 0.023*	*0.946± 0.019*		
	Recall	*0.913± 0.000*	*0.913± 0.000*		
	F1	*0.921± 0.011*	*0.929± 0.009*		

For each group: left column = Pub (trained without Amsterdam UMC weak data), right column = Pub+Amsterdam UMC-weak. Values are mean ± std across five random seeds

**Table 17:** Single-lead ablation on the 279 Amsterdam UMC expert segments with all of II, aVR, V1–V6 available (*n=279*)

**Class**	**Metric**	**II**		**aVR**	
		**Pub**	**+Weak**	**Pub**	**+Weak**
AF	Accuracy	*0.933± 0.003*	*0.920± 0.004*	*0.920± 0.007*	*0.922± 0.005*
	Precision	*0.841± 0.019*	*0.851± 0.038*	*0.798± 0.031*	*0.851± 0.026*
	Recall	*0.834± 0.023*	*0.752± 0.029*	*0.824± 0.039*	*0.759± 0.032*
	F1	*0.837± 0.008*	*0.797± 0.004*	*0.810± 0.017*	*0.801± 0.013*
SR	Accuracy	*0.861± 0.011*	*0.792± 0.012*	*0.862± 0.010*	*0.809± 0.019*
	Precision	*0.860± 0.021*	*0.757± 0.015*	*0.867± 0.016*	*0.771± 0.022*
	Recall	*0.919± 0.011*	*0.963± 0.012*	*0.909± 0.014*	*0.970± 0.007*
	F1	*0.888± 0.007*	*0.847± 0.007*	*0.887± 0.007*	*0.859± 0.012*

**Class**	**Metric**	**V1**		**V2**	
		**Pub**	**+Weak**	**Pub**	**+Weak**
AF	Accuracy	*0.918± 0.012*	*0.916± 0.003*	*0.918± 0.010*	*0.926± 0.014*
	Precision	*0.846± 0.011*	*0.818± 0.024*	*0.765± 0.039*	*0.806± 0.062*
	Recall	*0.738± 0.065*	*0.769± 0.029*	*0.876± 0.014*	*0.859± 0.031*
	F1	*0.787± 0.040*	*0.792± 0.009*	*0.816± 0.018*	*0.829± 0.026*
SR	Accuracy	*0.836± 0.005*	*0.831± 0.007*	*0.877± 0.008*	*0.864± 0.019*
	Precision	*0.818± 0.005*	*0.802± 0.008*	*0.882± 0.010*	*0.837± 0.022*
	Recall	*0.933± 0.015*	*0.952± 0.004*	*0.916± 0.013*	*0.959± 0.007*
	F1	*0.872± 0.005*	*0.871± 0.005*	*0.899± 0.007*	*0.894± 0.013*

**Class**	**Metric**	**V5**		**V6**	
		**Pub**	**+Weak**	**Pub**	**+Weak**
AF	Accuracy	*0.921± 0.004*	*0.919± 0.007*	*0.925± 0.004*	*0.927± 0.008*
	Precision	*0.819± 0.033*	*0.864± 0.005*	*0.818± 0.028*	*0.862± 0.011*
	Recall	*0.800± 0.038*	*0.724± 0.044*	*0.824± 0.055*	*0.772± 0.045*
	F1	*0.808± 0.010*	*0.787± 0.025*	*0.819± 0.017*	*0.814± 0.025*
SR	Accuracy	*0.865± 0.007*	*0.773± 0.037*	*0.845± 0.011*	*0.758± 0.027*
	Precision	*0.856± 0.008*	*0.735± 0.033*	*0.844± 0.009*	*0.722± 0.026*
	Recall	*0.931± 0.015*	*0.975± 0.003*	*0.910± 0.016*	*0.972± 0.011*
	F1	*0.892± 0.006*	*0.837± 0.021*	*0.876± 0.009*	*0.828± 0.014*

**Class**	**Metric**	**V3**		**V4**	
		**Pub**	**+Weak**	**Pub**	**+Weak**
AF	Accuracy	*0.934± 0.004*	*0.934± 0.007*	*0.948± 0.009*	*0.940± 0.003*
	Precision	*0.870± 0.023*	*0.874± 0.028*	*0.882± 0.017*	*0.901± 0.026*
	Recall	*0.803± 0.026*	*0.800± 0.060*	*0.866± 0.061*	*0.800± 0.036*
	F1	*0.835± 0.011*	*0.834± 0.025*	*0.872± 0.027*	*0.846± 0.011*
SR	Accuracy	*0.867± 0.008*	*0.827± 0.012*	*0.880± 0.008*	*0.811± 0.024*
	Precision	*0.847± 0.013*	*0.787± 0.015*	*0.874± 0.011*	*0.769± 0.026*
	Recall	*0.950± 0.005*	*0.976± 0.007*	*0.933± 0.014*	*0.980± 0.009*
	F1	*0.895± 0.005*	*0.871± 0.007*	*0.903± 0.007*	*0.861± 0.015*

For each column, all leads except the named one are zeroed *before* the ECG-FM encoder, so the representation is derived solely from that one lead. For each group: left column = Pub, right column = Pub+Amsterdam UMC-weak. Values are mean ± std across five random seeds

**Table 18:** Experiment 3 results: Classification performance of the fine-tuned ECG-FMs on public data only (Pub) and on public plus Amsterdam UMC-weak data (Pub+Amsterdam UMC-weak), reported for SR and AF on the second half of the Amsterdam UMC Expert test set (5–10 s segments), averaged across random seeds, standard deviation is given after ±

**Training setup**	**Class**	**Accuracy**	**Precision**	**Recall**	**F1**
Pub	AF	0.925±0.006	0.855±0.026	0.752±0.052	0.799± 0.021
	SR	0.887±0.005	0.911±0.010	0.905±0.012	0.908±0.004
Pub + Amsterdam UMC-weak	AF	0.934±0.006	0.851±0.045	0.812±0.025	0.830±0.009
	SR	0.894±0.003	0.894±0.008	0.940±0.010	0.916±0.003

**Table 19:** Experiment 3 results (Robustness check): first half (0–5 s) vs second half (5–10 s) of expert-annotated ECG recordings, for Pub (no weak data) and Pub+Amsterdam UMC-weak models. Mean and std across five random seeds; bootstrap 95% CIs pooled across seeds (10 000 resamples each)

Class	Metric	**Half 1 (0–5s)**		**Half 2 (5–10s)**		*Δ*	Ovlp	Cliff
		Mean±Std	95% CI	Mean±Std	95% CI			
**Pub**								
AF	Acc	*0.931± 0.006*	[0.907, 0.953]	*0.925± 0.003*	[0.902, 0.946]	*-0.005*	*+0.039*	*+0.72*
AF	Prec	*0.867± 0.026*	[0.786, 0.942]	*0.855± 0.035*	[0.766, 0.946]	*-0.013*	*+0.156*	*+0.40*
AF	Rec	*0.769± 0.052*	[0.648, 0.876]	*0.752± 0.021*	[0.661, 0.835]	*-0.017*	*+0.173*	*+0.12*
AF	F1	*0.814± 0.022*	[0.741, 0.876]	*0.799± 0.003*	[0.737, 0.854]	*-0.014*	*+0.113*	*+0.36*
SR	Acc	*0.894± 0.005*	[0.867, 0.919]	*0.887± 0.007*	[0.858, 0.916]	*-0.007*	*+0.049*	*+0.52*
SR	Prec	*0.917± 0.010*	[0.883, 0.950]	*0.911± 0.005*	[0.879, 0.941]	*-0.006*	*+0.057*	*+0.12*
SR	Rec	*0.911± 0.012*	[0.875, 0.945]	*0.905± 0.014*	[0.865, 0.941]	*-0.005*	*+0.067*	*+0.24*
SR	F1	*0.914± 0.004*	[0.890, 0.935]	*0.908± 0.007*	[0.882, 0.932]	*-0.006*	*+0.042*	*+0.52*
**Pub+Amsterdam UMC-weak**								
AF	Acc	*0.934± 0.006*	[0.910, 0.956]	*0.934± 0.006*	[0.910, 0.954]	*-0.000*	*+0.044*	*+0.12*
AF	Prec	*0.855± 0.045*	[0.742, 0.940]	*0.851± 0.042*	[0.742, 0.934]	*-0.004*	*+0.192*	*+0.16*
AF	Rec	*0.809± 0.025*	[0.723, 0.890]	*0.812± 0.027*	[0.723, 0.892]	*+0.003*	*+0.167*	*-0.08*
AF	F1	*0.830± 0.009*	[0.770, 0.882]	*0.830± 0.009*	[0.771, 0.881]	*-0.000*	*+0.110*	*+0.00*
SR	Acc	*0.899± 0.003*	[0.873, 0.924]	*0.894± 0.007*	[0.867, 0.921]	*-0.005*	*+0.047*	*+0.56*
SR	Prec	*0.903± 0.008*	[0.870, 0.935]	*0.894± 0.010*	[0.857, 0.927]	*-0.009*	*+0.057*	*+0.44*
SR	Rec	*0.938± 0.010*	[0.905, 0.965]	*0.940± 0.009*	[0.909, 0.966]	*+0.003*	*+0.056*	*-0.24*
SR	F1	*0.920± 0.003*	[0.898, 0.940]	*0.916± 0.005*	[0.893, 0.939]	*-0.003*	*+0.040*	*+0.52*

*Δ*= Half 2 minus Half 1 mean. Ovlp = CI overlap (negative = gap). Cliff = Cliff’s*Δ*;$|\text {Cliff}|\ge 0.474$= large effect.$^{\dagger }$Entries where CIs do not overlap (Ovlp*<0*) and the effect size is large$((|\text {Cliff}| \ge 0.474))$, indicating a clearly distinguishable, large effect favouring one of the sets

**Table 20:** Patient and positive-case counts by sex and age group in the Amsterdam UMC expert-labelled test set

**Age Group**	**Female**			**Male**			**Overall n**
	**n**	**AF**	**SR**	**n**	**AF**	**SR**	
<40	20	3	12	47	15	29	67
40-59	31	7	24	109	28	73	140
60-79	121	30	68	244	33	146	365
80+	13	0	9	7	1	4	20
Overall	185	40	113	407	77	252	592

*n* = total patients; AF and SR columns show number of positive cases for each class

**Table 21:** F1 score by sex and age group: Pub vs. Pub+Amsterdam UMC-weak

Age	Class	**Female**			**Male**			*Δ*	Ovlp	Cliff
		n	Mean±Std	95% CI	n	Mean±Std	95% CI			
**Pub**										
*<40*	AF	3	*0.000± 0.000*	[0.000, 0.000]	15	*0.896± 0.034*	[0.745, 0.994]	*-0.896*	*-0.745*	$-1.00^{\dagger }$
*<40*	SR	12	*0.895± 0.020*	[0.731, 1.000]	29	*0.975± 0.021*	[0.930, 1.000]	*-0.080*	*+0.070*	*-1.00*
40-59	AF	7	*1.000± 0.000*	[1.000, 1.000]	28	*0.819± 0.033*	[0.685, 0.920]	*+0.181*	*-0.080*	$+1.00^{\dagger }$
40-59	SR	24	*1.000± 0.000*	[1.000, 1.000]	73	*0.927± 0.006*	[0.877, 0.967]	*+0.073*	*-0.033*	$+1.00^{\dagger }$
60-79	AF	30	*0.930± 0.017*	[0.853, 0.985]	33	*0.684± 0.077*	[0.532, 0.806]	*+0.246*	*-0.047*	$+1.00^{\dagger }$
60-79	SR	68	*0.919± 0.012*	[0.864, 0.963]	146	*0.890± 0.012*	[0.850, 0.925]	*+0.029*	*+0.060*	*+0.84*
80+	AF	0	*0.000± 0.000*	[0.000, 0.000]	1	*0.000± 0.000*	[0.000, 0.000]	*+0.000*	*+0.000*	*+0.00*
80+	SR	9	*0.855± 0.030*	[0.659, 0.992]	4	*0.803± 0.057*	[0.422, 0.985]	*+0.052*	*+0.326*	*+0.68*
Overall	AF	40	*0.887± 0.016*	[0.803, 0.952]	77	*0.771± 0.044*	[0.688, 0.843]	*+0.116*	*+0.040*	*+1.00*
Overall	SR	113	*0.928± 0.004*	[0.890, 0.960]	252	*0.908± 0.007*	[0.881, 0.933]	*+0.020*	*+0.043*	*+1.00*
**Pub+Amsterdam UMC-weak**										
*<40*	AF	3	*0.000± 0.000*	[0.000, 0.000]	15	*0.889± 0.000*	[0.728, 1.000]	*-0.889*	*-0.728*	$-1.00^{\dagger }$
*<40*	SR	12	*0.909± 0.016*	[0.755, 1.000]	29	*0.993± 0.009*	[0.976, 1.000]	*-0.084*	*+0.024*	*-1.00*
40-59	AF	7	*1.000± 0.000*	[1.000, 1.000]	28	*0.867± 0.003*	[0.753, 0.951]	*+0.133*	*-0.049*	$+1.00^{\dagger }$
40-59	SR	24	*0.996± 0.010*	[0.985, 1.000]	73	*0.931± 0.006*	[0.884, 0.969]	*+0.065*	*-0.016*	*+1.00* $^{\dagger }$
60-79	AF	30	*0.916± 0.028*	[0.834, 0.976]	33	*0.743± 0.020*	[0.606, 0.853]	*+0.173*	*+0.019*	*+1.00*
60-79	SR	68	*0.924± 0.008*	[0.872, 0.966]	146	*0.895± 0.006*	[0.857, 0.929]	*+0.029*	*+0.057*	*+1.00*
80+	AF	0	*0.000± 0.000*	[0.000, 0.000]	1	*0.000± 0.000*	[0.000, 0.000]	*+0.000*	*+0.000*	*+0.00*
80+	SR	9	*0.849± 0.017*	[0.660, 0.992]	4	*0.857± 0.072*	[0.532, 0.985]	*-0.007*	*+0.325*	*-0.60*
Overall	AF	40	*0.862± 0.027*	[0.773, 0.932]	77	*0.812± 0.007*	[0.736, 0.876]	*+0.050*	*+0.103*	*+1.00*
Overall	SR	113	*0.931± 0.006*	[0.894, 0.962]	252	*0.915± 0.005*	[0.889, 0.939]	*+0.016*	*+0.046*	*+1.00*

Mean and std computed across five random seeds. Bootstrap 95% CIs resampled within each subgroup (10 000 resamples); wider CIs reflect smaller subgroup sizes (*n* = positive cases for the given class).*Δ*= Female minus Male mean F1. Ovlp = CI overlap (negative = gap between CIs). Cliff = Cliff’s*Δ*(Female vs. Male);$|\text {Cliff}|\ge 0.474$= large effect.$^{\dagger }$Entries where CIs do not overlap (Ovlp*<0*) and the effect size is large$((|\text {Cliff}| \ge 0.474))$, indicating a clearly distinguishable, large effect favouring one of the sex in the given age group

**Table 22:** Segment counts by signal quality for the Amsterdam UMC expert test set

**Subgroup**	**Total**	**AF**	**SR**
Clean	542	106	331
Noisy (Noisy but interpretable)	50	11	34
Poor quality (Too noisy to label)	8	-	-
Total	600	117	365

Noise classification uses the senior reviewer’s notes when available; otherwise, a segment is classified as noisy if at least one junior annotator mentioned noise or artifacts in their notes. Segments rated as uninterpretable are excluded from evaluation

**Table 23:** Model performance on the Amsterdam UMC expert test set stratified by signal quality

**Class**	**Metric**	**Pub**		**Pub+Amsterdam UMC-weak**	
		**Clean**	**Noisy**	**Clean**	**Noisy**
		$n_{\text {clean}}=542$,	$n_{\text {noisy}}=50$		
AF	Accuracy	*0.934± 0.006*	*0.892± 0.023*	*0.939± 0.007*	*0.884± 0.017*
	Precision	*0.868± 0.019*	*0.861± 0.107*	*0.860± 0.045*	*0.802± 0.065*
	Recall	*0.785± 0.056*	*0.618± 0.041*	*0.826± 0.020*	*0.636± 0.091*
	F1	*0.823± 0.025*	*0.717± 0.049*	*0.842± 0.013*	*0.705± 0.054*
SR	Accuracy	*0.895± 0.006*	*0.884± 0.017*	*0.900± 0.004*	*0.888± 0.011*
	Precision	*0.915± 0.011*	*0.938± 0.002*	*0.902± 0.008*	*0.908± 0.011*
	Recall	*0.913± 0.011*	*0.888± 0.025*	*0.938± 0.010*	*0.929± 0.016*
	F1	*0.914± 0.005*	*0.912± 0.014*	*0.920± 0.003*	*0.919± 0.008*

Values are mean ± std across five random seeds

## Appendix B Figures - misclassified examples

Figures 10–19 show examples of recordings that were misclassified by the model trained on public data (Pub, random seed 0).

## Appendix C Preliminary external evaluation on MIMIC continuous ECG data

To obtain an initial indication of out-of-institution performance on continuous ICU ECG, we constructed a small externally annotated test set from the MIMIC-III Waveform Database [39, 40]. We selected the first 20 adult patient folders listed on the PhysioNet website and retained only ECG records longer than 1 hour, as shorter recordings were frequently dominated by noise, yielding 110 eligible records. From each record, we then sampled a single 5-second segment, excluding the first and last 10 minutes to reduce the impact of start/end artefacts and noise. The annotation protocol matched that used for the Amsterdam UMC Expert dataset. In this preliminary exploratory analysis, each segment was labelled by a single senior clinician. The resulting dataset comprised 110 segments: 88 SR, 11 AF, 8 Other, and 3 segments judged too noisy to determine the underlying rhythm (Table 24).

**Table 24:** Detection performance on MIMIC for out-of-institution performance evaluation (mean ± std over 5 seeds)

**Training data**	**Class**	**Acc**	**Prec**	**Rec**	**F1**
Without weak data	AF	*0.946 ± 0.007*	*0.751 ± 0.041*	*0.709 ± 0.036*	*0.729 ± 0.035*
	SR	*0.916 ± 0.006*	*0.952 ± 0.008*	*0.945 ± 0.009*	*0.949 ± 0.004*
With weak data	AF	*0.938 ± 0.005*	*0.731 ± 0.030*	*0.636 ± 0.057*	*0.678 ± 0.033*
	SR	*0.884 ± 0.015*	*0.904 ± 0.016*	*0.961 ± 0.006*	*0.932 ± 0.008*

Models trained on PTB-XL + Chapman without and with Amsterdam UMC-weak

## Appendix D ECG-FM architecture and pretraining details

We use the open-weight ECG-FM of McKeen et al. and follow their released architecture [28]. ECG-FM adopts a wav2vec 2.0–style design [37] with a convolutional feature extractor that embeds raw ECG signals into latent representations, followed by a BERT-Base–like Transformer [38] encoder that captures contextual dependencies among these representations.

The feature extractor comprises four 1D convolutional blocks with 256 channels, kernel length 2, and stride 2, each followed by layer normalization and Gaussian Error Linear Unit (GELU) activation, producing a downsampled sequence of latent representations $z_t$. Relative positional embeddings are added to $z_t$, which are then passed to a Transformer encoder with 12 layers, 768-dimensional hidden states, 12 attention heads, and 3072-dimensional feed-forward layers, yielding contextualized representations $c_t$.

We rely on the ECG-FM pretrained weights obtained with the original hybrid self-supervised WCR objective, which combines wav2vec 2.0 to capture local temporal structure (i.e., intra-segment dynamics at each timestep), Contrastive Multi-Segment Coding (CMSC) to enforce global, clinically meaningful structure by encouraging similar representations for temporally adjacent segments from the same recording, and RLM to make the learned representations robust to missing or varying lead configurations.
